# α-catenin interaction with YAP/FoxM1/TEAD-induced CEP55 supports liver cancer cell migration

**DOI:** 10.1186/s12964-023-01169-2

**Published:** 2023-06-28

**Authors:** Yingyue Tang, Lena Thiess, Sofia M. E. Weiler, Marcell Tóth, Fabian Rose, Sabine Merker, Thomas Ruppert, Peter Schirmacher, Kai Breuhahn

**Affiliations:** 1 Institute of Pathology, University Hospital Heidelberg, Heidelberg, Germany; 2 CFMP, Core Facility for Mass Spectrometry & Proteomics at the Center for Molecular Biology (ZMBH), Heidelberg University, Heidelberg, Germany

**Keywords:** Hippo pathway, Yes-associated protein, Forkhead box M1, TEA domain transcription factors, Adherens junctions, Cell–cell contact, BioID assay

## Abstract

**Background:**

Adherens junctions (AJs) facilitate cell–cell contact and contribute to cellular communication as well as signaling under physiological and pathological conditions. Aberrant expression of AJ proteins is frequently observed in human cancers; however, how these factors contribute to tumorigenesis is poorly understood. In addition, for some factors such as α‐catenin contradicting data has been described. In this study we aim to decipher how the AJ constituent α‐catenin contributes to liver cancer formation.

**Methods:**

TCGA data was used to detect transcript changes in 23 human tumor types. For the detection of proteins, liver cancer tissue microarrays were analyzed by immunohistochemistry. Liver cancer cell lines (HLF, Hep3B, HepG2) were used for viability, proliferation, and migration analyses after RNA*interference*-mediated gene silencing. To investigate the tumor initiating potential, vectors coding for α‐catenin and myristoylated AKT were injected in mice by hydrodynamic gene delivery. A BioID assay combined with mass spectrometry was performed to identify α‐catenin binding partners. Results were confirmed by proximity ligation and co-immunoprecipitation assays. Binding of transcriptional regulators at gene promoters was investigated using chromatin-immunoprecipitation.

**Results:**

α‐catenin mRNA was significantly reduced in many human malignancies (e.g., colon adenocarcinoma). In contrast, elevated α‐catenin expression in other cancer entities was associated with poor clinical outcome (e.g., for hepatocellular carcinoma; HCC). In HCC cells, α‐catenin was detectable at the membrane as well as cytoplasm where it supported tumor cell proliferation and migration. In vivo, α‐catenin facilitated moderate oncogenic properties in conjunction with AKT overexpression. Cytokinesis regulator centrosomal protein 55 (CEP55) was identified as a novel α‐catenin-binding protein in the cytoplasm of HCC cells. The physical interaction between α‐catenin and CEP55 was associated with CEP55 stabilization. CEP55 was highly expressed in human HCC tissues and its overexpression correlated with poor overall survival and cancer recurrence. Next to the α‐catenin-dependent protein stabilization, CEP55 was transcriptionally induced by a complex consisting of TEA domain transcription factors (TEADs), forkhead box M1 (FoxM1), and yes-associated protein (YAP). Surprisingly, CEP55 did not affect HCC cell proliferation but significantly supported migration in conjunction with α‐catenin.

**Conclusion:**

Migration-supporting CEP55 is induced by two independent mechanisms in HCC cells: stabilization through interaction with the AJ protein α‐catenin and transcriptional activation via the FoxM1/TEAD/YAP complex.

**Supplementary Information:**

The online version contains supplementary material available at 10.1186/s12964-023-01169-2.

## Abstract (plain language summary)

Cell–cell contact in epithelial cells is important for cell polarity, cellular compartmentalisation, as well as tissue architecture during development, homeostasis, and regeneration of adult tissues in metazoans. In this context, adherens junctions (AJs) mechanically sense cell contact information with direct impact on cytoskeletal remodelling, the regulation of signalling pathways, and eventually cell biology. Indeed, the loss of cell–cell contact and cellular polarity are key features in human carcinogenesis and important pathological parameters for the identification of many epithelial tumors.

We demonstrate in this study, that overexpression of the AJ constituent α‐catenin is frequently observed in human hepatocellular carcinoma (HCC). α‐catenin supports HCC cell proliferation and migration. Together with the oncogene AKT, α‐catenin moderately facilitates tumor initiation in mouse livers. Using mass spectrometry, we identified several new α‐catenin interaction partners in the cytosol of liver cancer cells, including the cytokinesis regulator centrosomal protein 55 (CEP55). CEP55 mediates pro-migratory effects and its overexpression in HCC cells is controlled by two molecular mechanisms: α‐catenin-dependent protein stabilization and transcriptional induction by the TEA domain transcription factors (TEADs)/forkhead box M1 (FoxM1)/yes-associated protein (YAP) complex.

In summary, we here describe a new mechanism how changes in cell–cell contact support liver cancer formation and progression. This study demonstrates that dysregulation of the AJ component α‐catenin contributes to liver carcinogenesis via distinct molecular mechanisms.

Video Abstract

**Supplementary Information:**

The online version contains supplementary material available at 10.1186/s12964-023-01169-2.

## Background

Adherens junctions (AJs), desmosomes, tight junctions, as well as gap junctions facilitate different types of cell–cell contact and contribute to e.g., cell polarity, spatial organization of cells in tissues, formation of physical barriers, and communication [1]. AJs consist of transmembrane cadherins, which form a protein complex with p120, β-catenin, and α-catenin to physically connect AJs with the cytoskeletal network [1]. However, this ‘linear’ picture is probably an over-simplification as more than 170 colocalizing proteins may affect AJ dynamics as well as their function [2].

Depending on the presence of cell–cell contact and physical forces, AJs can control different cellular processes such as proliferation, differentiation, and migration under physiological and pathological conditions. Regarding these processes, two different mechanisms have been described. First, sequestration of transcriptionally active proteins at the junctional complex as illustrated for the transcriptional activators β-catenin and yes-associated protein (YAP) [3, 4]. Second, AJs interact with actin filaments via α-catenin and with microtubule filaments via i.e., dynein, and therefore contribute to cell motility and mitosis, respectively [5, 6].

Due to their relevance in cell homeostasis and their impact on various cell functions, expression changes or mutations of AJ proteins are associated with the development and progression of cancer [1]. For example, the deletion of E-cadherin and simultaneous activation of Kras increased tumor formation in vivo compared to Kras activation alone [7]. Vice versa, upregulation of other AJ components is associated with tumor aggressiveness as illustrated for N-cadherin in prostate cancer cells [8]. Indeed, the gradual replacement of E-cadherin by N-cadherin and associated tumor-supporting signaling changes have been described for different tumor types [9].

Interestingly, individual AJ constituents can facilitate tumor-promoting or tumor-suppressive properties in different cancer entities. For example, increased cadherin-11 expression is associated with metastasis of prostate cancer to the bone, while promoter CpG methylation and silencing of the CDH11 gene in hepatocellular carcinoma (HCC) and other tumor types is associated with tumor-supporting cell properties [10]. In addition, the atypical cadherin family member FAT1 is differentially expressed in human cancer types and can promote (e.g., cervical cancer) or inhibit (e.g., liver cancer) tumor cell migration [11, 12].

An important example for an AJ-associated factor with a controversial role in tumorigenesis is α‐catenin (also known as αE‐catenin). For example, α‐catenin is down-regulated in many human cancers through genomic losses of chromosome 5q, epigenetic inactivation of the CTNNA1 gene, or possibly post-translational modifications [13]. Depletion of α‐catenin stimulates cell motility and proliferation via activation of ERK signaling and supports the nuclear enrichment of the Hippo pathway effector YAP [4, 14]. Indeed, most studies suggest a tumor-suppressive function of this protein; however, the expression of α‐catenin has been controversially discussed as its up- and downregulation were described for some tumor types. For example, decreased and also increased α‐catenin expression was shown for colorectal cancer while a mixed expression pattern was published for gastric or pancreatic cancers [15]. For HCC, most studies describe diminished expression of α‐catenin; however, a positive correlation of α‐catenin expression with HCC dedifferentiation was also shown [16, 17]. To our knowledge, functional in vitro or in vivo analyses on α‐catenin in liver cancer cells, which could shed a light on its potential pro- or antitumorigenic properties in hepatocarcinogenesis, are missing. However, the in part conflicting results from different tumor tissues suggest that the structural reorganization of AJs or induction/repression of distinct junctional proteins does not stereotypically support or suppress tumor formation and progression. Instead, the expression and functional relevance of AJ complex constituents must be individually evaluated in different cancer entities to decipher their role as tumor-suppressor or tumor-supporting factors and oncogenes.

In this study, we investigate how α‐catenin contributes to liver cancer initiation and progression. Systematic expression analyses at the transcript and protein levels illustrate that α‐catenin overexpression in HCC cells is associated with poor patient survival. α‐catenin supports HCC cell proliferation as well as migration and exerts moderate oncogenic properties in conjunction with the serine-threonine protein kinase AKT. The centrosomal protein 55 (CEP55) is identified as a novel cytoplasmic α‐catenin interaction partner, which stimulates migration but not mitosis. Overexpression of CEP55 in human HCCs correlates with worse clinical outcome. Enrichment of CEP55 depends on α‐catenin-mediated protein stabilization and transcriptional induction via a complex containing YAP, TEA domain transcription factors (TEADs), and forkhead box M1 (FoxM1). Thus, our study delineates a new functional aspect of how the aberrant expression of the AJ protein α‐catenin contributes to liver carcinogenesis.

## Methods

Detailed information on used buffers, nucleic acid sequences (primers, siRNAs), and antibodies are listed in Suppl. Tables 1–6.

### Vectors

For generation of the pDEST-Flag-N-CTNNA1 and pBirA-Flag-N-CTNNA1 vectors, human CTNNA1 (NM_001903.5, transcript variant 1) flanked with attB sequences was amplified by PCR from cDNA prepared from HLF cells using a proof-reading polymerase (Phusion® High-Fidelity DNA Polymerase, New England Biolabs, Frankfurt am Main, Germany). CTNNA1 cDNA was transferred into the pDONR201 vector utilizing the Gateway™ BP Clonase™ II Enzyme mix followed by the transfer into respective destination vectors using the Gateway™ LR Clonase™ II Enzyme mix (Thermo Fisher Scientific, Darmstadt, Germany).

For hydrodynamic gene delivery, human CTNNA1 was inserted into the pT3-EF1a-fullMCS-IRES-GFP vector using the restriction sites BglII and EcoRI. pT3-EF1ɑ-myrAKT (10 μg), pT3-EF1ɑ-CTNNA1-IRES-GFP (10 μg), pT3-EF1ɑ-YAPS127A-IRES-GFP (10 µg), pT3-EF1ɑ-β-catenin-IRES-GFP (10 µg), and Sleeping Beauty transposase (SB, 2 μg) were injected as previously described [18]. myr-AKT was used as co-injected oncogene in this study as it has been shown to facilitate mild oncogenic properties in liver cancer [19].

All vector constructs were validated by sequencing (Seqlab-Sequence Laboratories, Göttingen, Germany). Cloning primers are listed in Suppl. Table 2.

### Cell culture and genetic manipulation

The hepatocyte-derived cell lines HLF, and HLE (Japanese Collection of Research Bioresources; JCRB, Osaka, Japan), HepG2, Hep3B, HHT4, Huh1, Huh6, Huh7, and SNU182 (LGC Standards, Wesel, Germany) were cultured in DMEM, RPMI, or MEM medium (Sigma Aldrich, Steinheim, Germany) supplemented with 10% fetal bovine serum and 1% penicillin/streptomycin. All cell lines were maintained at 37°C and 5% CO_2_ in a humidified atmosphere. Cells were routinely tested for mycoplasma contamination and authentication was performed by short tandem repeat analysis (DSMZ, Braunschweig, Germany).

For transient transfection of gene-specific small interfering RNA (siRNA, Microsynth, Göttingen, Germany), Oligofectamine or Lipofectamine RNAiMax were used according to the manufacturer’s protocols (Thermo Fisher Scientific). siRNAs were used at a final concentration of 20 or 40 nM. For transfection, cells were seeded into 6-well plates one day prior to transfection. Equimolar concentrations of nonsense siRNA-transfected cells were used as negative controls (scrambled siRNA; scr.). Cell culture medium was replaced after 24 h and cells were investigated after the indicated time points. siRNAs used in this study are listed in Suppl. Table 3.

Transfection of plasmids was performed using Fugene HD transfection reagent according to the manufacturer’s instructions (Promega, Mannheim, Germany).

To produce lentiviral particles in HEK293T cells, plasmids containing human CTNNA1 cDNA (pBirA-Flag-N-CTNNA1), pMD2G, and psPAX2 vectors were transfected using polyethylenimine (Addgene plasmids pMD2.G #12,259 and psPAX2 #12,260). Cells were infected with virus-containing supernatant overnight. After that, cell culture medium was replaced with medium containing antibiotics for selection of cells with stable vector integration (1 µg/ml puromycin).

### Quantitative real-time PCR (qPCR)

The NucleoSpin RNA II kit was used for isolation of total RNA according to the manufacturer’s protocol (Macherey–Nagel, Düren, Germany). Reverse transcription was done using 500 ng of total RNA (Takara, Shiga, Japan). qPCR reactions were performed utilizing the ABsolute qPCR SYBR Green ROX Mix (Steinbrenner, Wiesenbach, Germany) with the following cycling conditions: 95°C for 15 min, followed by 40 cycles of 95°C for 15 s and 60°C for 60 s (Quant Studio 3 real-time PCR system; Applied Biosystems, Darmstadt, Germany). Subsequent melting curve analysis was applied to assure product specificity (95°C for 15 s, 60°C for 30 s, 60–95°C with Δ0.5°C/sec). For liver cancer cell lines, β2-microglobulin (B2M) and glyceraldehyde-3-phosphate dehydrogenase (GAPDH) were used as housekeeping genes for normalization. Primers used for real-time PCR are listed in Suppl. Table 4.

### Western immunoblotting

Total protein extracts were isolated by using 10 × Cell Lysis Buffer (Cell Signaling/New England Biolabs, Cambridge, UK) supplemented with 1 × Protease Inhibitor Cocktail (Sigma, St. Louis, USA). Protein concentrations were measured using the Bradford protein assay (Bio-Rad, California, USA). Thirty to 50 µg of total protein extracts were separated using 8 to 12% sodium dodecyl sulfate–polyacrylamide gel electrophoresis (SDS-PAGE) followed by electro-transfer of proteins to a nitrocellulose membrane. After blocking the membrane with Tris-buffered saline/Tween 20 (TBST) containing 5% milk or bovine serum albumin, primary antibodies were added and the membrane was incubated at 4°C overnight. After washing, the appropriate secondary antibodies (1:20,000; IRDye 680 and 800, LiCor Biosciences, Bad Homburg, Germany) were added and incubated at room temperature for 1 h. Signal detection and visualization were performed with the Odyssey-CLx Infrared Imaging system and the ImageStudio Lite software (LiCor Biosciences). Antibodies used for Western Immunoblotting are listed in Suppl. Table 5.

### Co-immunoprecipitation (Co-IP)

For Co-IP experiments, cells were seeded on 10 cm dishes and harvested with Co-IP lysis buffer containing 1 × Protease Inhibitor Cocktail, 1 mM PMSF, and 1 mM DTT 48 h after transfection. Dynabeads Protein G (Thermo Fisher Scientific) were washed once with 50 µl of a 50 mM Glycine solution (pH 2.8) at room temperature for 5 min. After removal of liquid utilizing the magnetic separator (Qiagen, Hilden, Germany), 2 µg specific primary antibodies diluted in PBST were added to the beads followed rotation/incubation at 4°C for 1.5 h. Using the magnetic separator, beads were washed twice. Next, 1 to 2 mg of protein derived from cells were carefully mixed with the beads, normalized to 1 ml with Co-IP lysis buffer, and incubated at 4°C overnight. The protein/antibody-bead complexes were rinsed with PBS four times and denatured with Laemmli buffer. After mixing at 500 rpm at room temperature for 20 min, samples were heated at 95°C for 8 min and analyzed by Western immunoblotting. Antibodies used for Co-IP experiments in this study are listed in Suppl. Table 5.

### Chromatin immunoprecipitation (ChIP)

The ChIP assay was conducted as previously described [20]. Cells were fixed with formaldehyde, harvested with ice-cold RIPA buffer supplemented with 1 × Protease Inhibitor Cocktail, and sonicated (S-4000 Sonicator, Qsonica, Newton, USA).

For preclearing of protein lysates, Dynabeads Protein G (Thermo Fisher Scientific) were washed twice and resuspended in RIPA buffer followed by incubation with cell lysates at 4°C for 1.5 h with rotation. For blocking, beads were prepared by washing with RIPA buffer and resuspended in RIPA followed by blocking with BSA (1 mg/ml) and salmon sperm DNA (0.3 mg/ml; Invitrogen, Carlsbad, USA) at 4°C with rotation for 1.5 h. Cell lysates and 2 µg of antibody were added to the beads followed by incubation at 4°C with rotation overnight. Beads were washed with RIPA buffer, IP wash buffer using the magnetic separator and DNA was eluted with Talianidis elution buffer at 65°C for 10 min. Reversal of crosslinking was achieved by adjusting the samples to 0.2 mol/l NaCl and incubation at 65°C for 5 h. Purification of DNA was performed using the Nucleospin® Gel and PCR Clean-up kit (Machery-Nagel) according to the manufacturer’s protocol. Abundance of genomic sequences was analyzed with qPCR using a serial dilution of genomic DNA as reference. Antibodies and primers used in our study are listed in Suppl. Tables 5 and 6.

### Immunofluorescence

Cells were seeded on 18 mm coverslips one day before staining. Fixation was done in 4% formaldehyde for 15 min or precooled methanol for 5 min followed by acetone treatment for 1 min. After permeabilization with 0.2% Triton X-100/PBS for 5 min, slides were washed two times with PBS and blocked in 1% BSA/PBS at room temperature for 30 min. Primary antibodies diluted in PBS were added and incubated at 4°C overnight. After washing, secondary antibodies were added and incubated at room temperature for 1 h. Slides were washed with PBS three times, shortly washed with water, and then dried and mounted with DAPI-containing Fluoromount G (Southern Biotech, Birmingham, USA). Fluorescence microscopy and digital documentation was performed using either a HAL 100 microscope (Zeiss, Jena, Germany) or an Olympus IX81 microscope (Olympus, Hamburg, Germany).

### Functional assays

For measuring viability and apoptosis, cells were seeded on 6-well plates and transfected with siRNAs. Resazurin reagent (R&D Systems, Minneapolis, USA) or CellTox™ Green reagent (CellTox™ Green Cytotoxicity Assay, Promega, Mannheim, Germany) were added according to the manufacturer’s protocol. Results were acquired using a Fluostar Omega microplate reader (BMG Labtech, Ortenberg, Germany). For viability assessment, an excitation wavelength of 544 nm and an emission wavelength of 590 nm were used. For apoptosis assessment, fluorescence was measured at an excitation wavelength of 485 nm and an emission wavelength of 520 nm.

Proliferation was analyzed using a BrdU-ELISA assay 96 h after transfection (Cell proliferation ELISA Biotrak, GE Healthcare/Amersham, Freiburg, Germany). Signals were measured at 450 nm using a Fluostar Omega microplate reader.

For measuring colony formation, 1,000 cells were seeded in 6-well plates. Cells formed colonies under full medium conditions within two weeks. Colonies were washed with PBS one time and stained with 0.5% crystal violet at room temperature for 30 min. After a final washing step with water, plates were dried and results were digitally documented (Bio-Techne, Minneapolis, MN, USA). Colony density was quantified using the ImageJ software [21].

For the analysis of lateral cell migration, 15,000 cells were seeded in each side of an ibidi culture insert 24 h after siRNA transfection (ibidi, Martinsried, Germany). The next day, cells were treated with 0.5 or 5 μg/ml mitomycin-C for 3 h to prevent proliferation (Pharmacy, University Hospital Heidelberg, Germany). The ibidi inserts were carefully removed to produce defined gaps of 500 μm between two cell populations. Directional cell migration was monitored by taking three pictures for each gap at different time points. Acquisition was done with an Olympus CKX41 microscope using the Olympus CellSens Dimension software (Olympus). The gap area was measured using the ImageJ software and relative cell migration was determined by calculating the difference of cell-free areas at different time points.

### Proximity ligation assay (PLA)

The DuoLink in situ PLA was performed according to the manufacturer’s instructions (Sigma-Aldrich). Briefly, cells were seeded on 18 mm glass coverslips one day prior to the staining. Cells were washed three times with PBS containing 2 mM MgCl_2_ and fixed with 4% paraformaldehyde at room temperature for 10 min. Fixed cells were washed four times with PBS for 5 min, permeabilized with 0.2% Triton X-100 in PBS for 5 min, and washed again twice with PBS for 5 min. Next, cells were blocked with Blocking solution for 30 min at room temperature and incubated with the primary antibodies diluted in Antibody Diluent at 4°C overnight. Subsequently, cells were washed twice with Wash Buffer A and incubated with pre-diluted Minus and Plus PLA probes/complementary oligonucleotides in Antibody Diluent at 37°C for 1 h. Cells were washed twice with Wash Buffer A and then incubated with ligation solution at 37°C for 30 min. After ligation, samples were again washed twice with Wash Buffer A for 2 min at room temperature and incubated with amplification-polymerase solution at 37°C for 100 min. Finally, cells were washed twice with Wash Buffer B at room temperature for 10 min, washed once with 0.01X Wash Buffer B at room temperature for 1 min, and coverslips were mounted on the slide with DAPI Fluoromount-G mounting medium. Fluorescence images were captured using an Olympus IX81 microscope. Antibodies used in our study are listed in Suppl. Tables 5.

### Immunohistochemistry staining and HCC tissue microarrays

Formalin-fixed, paraffin embedded (FFPE) tissue sections were cut into 2 µm thick sections and mounted onto microscope slides. Samples were deparaffinized with xylene three times for 5 min, followed by rehydration in 100% ethanol twice for 2 min, 96% ethanol for 2 min, and 70% ethanol for 2 min twice. Tissues were rinsed with distilled water. Antigen retrieval was achieved by either steamer or pressure cooker with target retrieval solution (DAKO, Hamburg, Germany). Tissue sections were washed with TBST and incubated with the primary antibody diluted in antibody diluting buffer (DAKO) overnight. After three washing steps with TBST (5 min each), either the secondary biotin-conjugated antibody or the Enhancer Detection Line was applied for 30 min (DCS, Hamburg, Germany). Followed by three washing steps in TBST, the streptavidin horseradish peroxidase (HRP, DAKO) or alkaline phosphatase (AP)-Polymer detection line (DCS) were used, followed by chromogen development using AP-Red (Zytomed, Berlin, Germany), aminoethylcarbazole (AEC) (DAKO), or DAB (DAKO), respectively. Antibodies used for immunohistochemistry are listed in Suppl. Table 5.

The analysis of patient material in this study was approved by the institutional ethics committee of the Medical Faculty of the University of Heidelberg (approval number: S-376/2018). The HCC tissue microarray used in this study contained 216 non-malignant liver tissues, 9 Dysplastic Nodules, and 470 HCCs (lower case numbers in this study are caused by the loss of tissues in the staining process). For the semi-quantitative analysis of individual immunohistochemical stains, both quantity and intensity of signals were evaluated. Quantity was scored as follows: 0 = no expression, 1 = up to 1% of cells, 2 = 1–9% of cells, 3 = 10–50% of cells, 4 > 50% of cells. Staining intensity was scored from 0 to 3 (0 = negative, 1 = low, 2 = medium, and 3 = strong). Both quantitative and qualitative values were multiplied resulting in a score ranging from 0 to 12, which was used for further statistical analysis.

### Cancer patient expression data

The Cancer Genome Atlas (TCGA) data for 23 cancer types and respective non-malignant tissues was analyzed for α‐catenin expression using the University of Alabama at Birmingham Cancer data analysis portal (UALCAN) [22]. In addition, a previously published independent HCC cohort was analyzed [23].

TEAD4 and FoxM1 ChIP-Seq data sets from HepG2 cells were obtained from the ENCODE project (GSE170161 and GSE169998, respectively) [24]. YAP ChIP-Seq data from NCI-H2052 cells was retrieved from GEO (GSE61852) [25]. ChIP-Seq data was visualized using the R package trackplot [26].

### Proximity-dependent labeling and mass spectrometry

After generating the stable cell lines with inducible overexpression of N-terminal tagged BirA CTNNA1 and the empty BirA vector, cells were seeded on 15 cm dishes and treated with 1 µg/ml Doxycycline for 48 h and 50 µM biotin for 24 h. Proteins were collected with Cell Lysis buffer, sonicated (S-4000 Sonicator) and centrifuged at 4°C with 16,500 rpm for 10 min. Streptavidin magnetic beads (Dynabeads® MyOne™ Streptavidin C1, Thermo Fisher) were prepared by washing with BioID lysis buffer twice. Protein lysates were added to the beads followed by incubation under rotation at 4°C overnight. The bead/protein complexes were collected using a magnetic separator followed by five washing steps. After the final washing step, biotinylated proteins were separated from beads with Laemmli buffer saturated with biotin at 95°C for 10 min. Samples were subjected to SDS-PAGE electrophoresis and stained with colloidal Coomassie Brilliant Blue G250.

Proteins were reduced and alkylated by incubating gel pieces with 60 µL dithiothreitol (40 mM) in 50 mM tetraethylammonium tetrahydroborate buffer (TEAB, pH 8.5) at 57°C for 30 min followed by incubation with 60 µl iodoacetamide (59 mM) in 50 mM TEAB at 25°C in the dark for 20 min. After dehydration with 60 µl 100% acetonitrile (ACN), 30 µl of trypsin (8 ng/µl in 50 mM TEAB) was added and incubated at 37°C overnight. After adding 20 µl of 0.1% trifluoroacetic acid, peptides were extracted by dehydration two times in 20 µl ACN and 30 µl 50 mM TEAB for 20 min. Collected supernatants were dried by vacuum. For high-performance liquid chromatography (HPLC)-mass spectrometry (MS) analysis (Ultimate 3000 coupled to an Orbitrap QE HF, Thermo Fischer Scientific), samples were dissolved in 15 µl 0.1% TFA and loaded to an in-house packed analytical column (inner diameter 75 µm × 20 cm; CS-Chromatographie Service) with a flow rate of 550 nl/min. Peptides were separated using a linear gradient (3–40% of solvent B) for 60 min at 300 nl/min using solvent A (0.1% formic acid (FA)/1% ACN) and solvent B (0.1% FA, 10% water, 89.9% ACN). The mass spectrometer was operated in data-dependent acquisition mode, automatically switching between MS and MS2. MS spectra (m/z 400–1,600) were acquired in the Orbitrap at 60,000 (m/z 400) resolution. Fragmentation in HCD cell was performed for up to 15 precursors and MS2 spectra were acquired at 15,000 (m/z 400) resolution. Raw files were processed using MaxQuant version 1.6.12.0 [27]. MS2 spectra were searched against the Uniprot human proteome database (UP000005640_9606.fasta downloaded Nov 2019) and the contaminants database provided together with software using the following parameters: carbamidomethylation of cysteine residues as fixed modification and acetyl (Protein N-term), oxidation (M) and deamidation (Q,N) as variable modifications. Trypsin/P as the proteolytic enzyme with up to 2 missed cleavages was allowed. The maximum false discovery rate for proteins and peptides was set to 0.01 and a minimum peptide length of 7 amino acids was required. All other parameters were default parameters of MaxQuant. LFW values were calculated by MaxQuant and used for further data analysis. The mass spectrometry proteomics data have been deposited to the ProteomeXchange Consortium via the PRIDE partner repository (https://www.ebi.ac.uk/pride/; PXD039222).

### Mouse experiments

The experimental setup was approved by the German Regional Council of Baden-Wuerttemberg (ref. number: G-187/19; Karlsruhe, Germany). All experiments were performed in accordance with the institutional regulations of the IBF (Interfakultäre Biomedizinische Forschungseinrichtung, University of Heidelberg) under pathogen-free (SPF) conditions. The mouse colony was housed under a 12 h light/dark cycle with free access to water and food. Exclusion and termination criteria were defined in the ATBW criteria. Hydrodynamic tail vein injection was performed as previously described [18]. Livers were isolated 12 weeks after vector injection as myr-AKT alone usually does not cause tumor formation at this time point [19]. At indicated time points, mouse livers were isolated, digitally documented, and stored in liquid nitrogen or buffered formalin for further analysis.

### Software and statistics

Data are presented as mean ± standard deviation. The Spearman’s rank correlation coefficient (r) was used as a statistical measure of association. Overall survival was analyzed by the Kaplan-Meyer method using the Log-rank (Mantel-Cox) test. Statistical comparison between two groups was performed using the non-parametric Mann–Whitney U test or unpaired t test. All statistical analyses were performed using Prism 8 (GraphPad Prism Software, San Diego, USA). Significance levels were defined as: p* ≤ 0.05, p** ≤ 0.01, and p*** ≤ 0.001. Cutoff Finder was used as previously described [28].

## Results

### α‐catenin is overexpressed in human HCCs

For most tumor types, downregulation of α‐catenin has been described, which points to a tumor-suppressive role of this AJ-associated protein [15]. By analyzing publicly available cancer patient data, we confirmed this reduction of α‐catenin at transcript levels for 6/23 human tumor types in comparison to respective non-malignant tissues [29]. For example, significantly lower amounts of α‐catenin mRNA were detectable for lung squamous cell carcinoma (LUSC) and colon adenocarcinoma (COAD) (Suppl. Figure S1A). Interestingly, for other cancers (8/23) a significant α‐catenin induction was detectable as illustrated for liver hepatocellular carcinoma (HCC/LIHC) and cholangiocarcinoma (CHOL) (Suppl. Figure S1A, B). For 9 tumor types no significant transcript changes were observed (e.g., lung adenocarcinoma; LUAD) (data not shown). As expression of α‐catenin has been controversially discussed in human hepatocarcinogenesis, we further investigated the transcript and protein abundance of α‐catenin in different HCC cohorts.

**Fig. 1:**
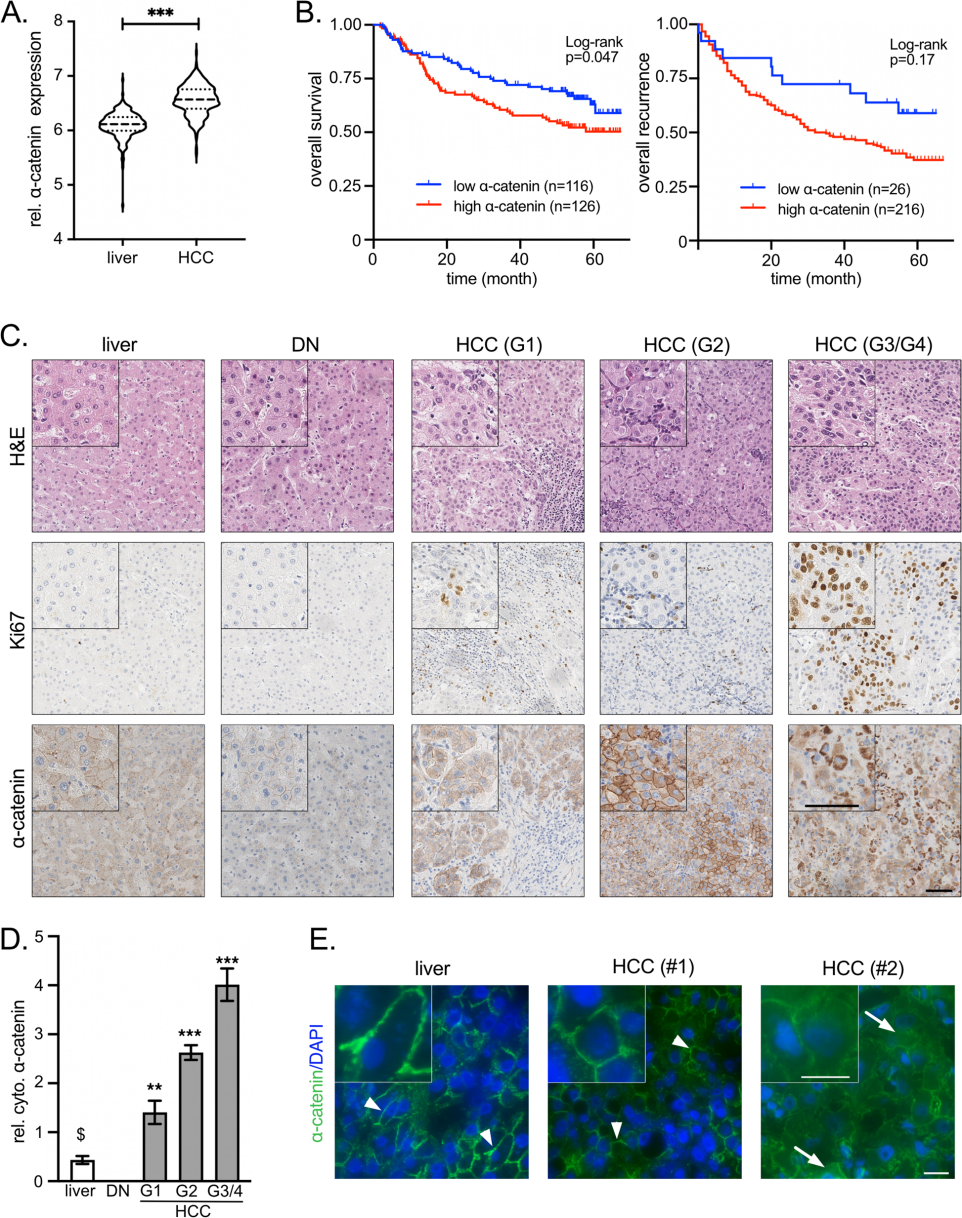
Expression of α‐catenin mRNA and protein in human HCCs. **A** Comparison of α-catenin transcriptome data derived from human HCC tissues and adjacent liver tissues [23]. In total, 242 HCC tissues and 239 nontumorous liver tissues were included in this analysis. Statistical test: Mann–Whitney U test. ***p ≤ 0.001. **B** Kaplan–Meier plots showing HCC patient survival and tumor recurrence in relation to α-catenin mRNA expression. Patients were divided in two groups with low and high α-catenin expression using Cutoff Finder. Statistical test: Log-rank test. *p*-values and group sizes are indicated. **C** Representative images of hematoxylin and eosin (H&E) and immunohistochemistry stains for Ki67 and α‐catenin. The HCC tissue microarray (*n* = 695) contains normal livers (*n* = 216), DNs (*n* = 9) and HCCs (*n* = 470). The group of HCCs consists of well differentiated tumors (G1 and G2; *n* = 86 and 309, respectively) and poorly differentiated tumors (G3/4; *n* = 75). Scale bars: 60 µm. **D** Bar graph summarizing the distribution of cytoplasmic α‐catenin positivity in normal livers, DNs, and HCCs (G1, G2, and G3/4). Statistical test: Mann–Whitney U test. $: normal livers were used for statistical comparison. **p ≤ 0.01, ***p ≤ 0.001. **E** Immunofluorescence images of normal livers and HCCs stained for α‐catenin. Nuclei are visualized by DAPI. Membranous α‐catenin (arrow heads) and cytoplasmic α‐catenin (arrow) are indicated. Three normal livers and 5 HCCs were investigated. One exemplary normal liver and two HCCs with prominent membranous (#1) and cytoplasmic (#2) α‐catenin positivity are shown. Scale bar: 20 µm

First, higher α‐catenin mRNA abundance compared to non-malignant livers was validated in an independent HCC patient cohort (Fig. 1A) [23, 30]. For both analyzed cohorts, overexpression of α‐catenin statistically associated with worse overall patient survival but not with tumor recurrence (Fig. 1B, Suppl. Figure S1C). Second, immunohistochemical stains of HCC tissue micro-arrays containing normal liver tissues, premalignant lesions (Dysplastic Nodules, DNs), and HCCs revealed that α‐catenin was moderately expressed at the membrane of hepatocytes (Fig. 1C). However, we observed elevated α‐catenin expression at the membrane and in the cytoplasm of HCC cells in about 43% and 59% of all cases, respectively. Especially for poorly differentiated HCC (G3/G4), a pronounced increase of cytoplasmic α‐catenin was detectable (Fig. 1D, Suppl. Figure S1D). For the accumulation of α‐catenin in the cytoplasm, a moderate but significant positive correlation with the proliferation marker Ki67 was observed in the group of HCCs (*r* = 0.33, *p* ≤ 0.01). Interestingly, a prominent α‐catenin expression was exclusively detectable in HCCs but not in DNs, suggesting that the observed dysregulation is a feature of and not causative for malignant transformation. Lastly, membranous expression and also cytoplasmic localization of α‐catenin was confirmed in a smaller cohort of HCCs by immunofluorescence (Fig. 1E).

The results demonstrate that α‐catenin is overexpressed in a subgroup of HCC tissues. In HCC cells, α‐catenin is localized at the membrane but can also be found in the cytoplasmic compartment.

### α‐catenin is a moderate oncogene and supports liver cancer cell viability and migration

**Fig. 2:**
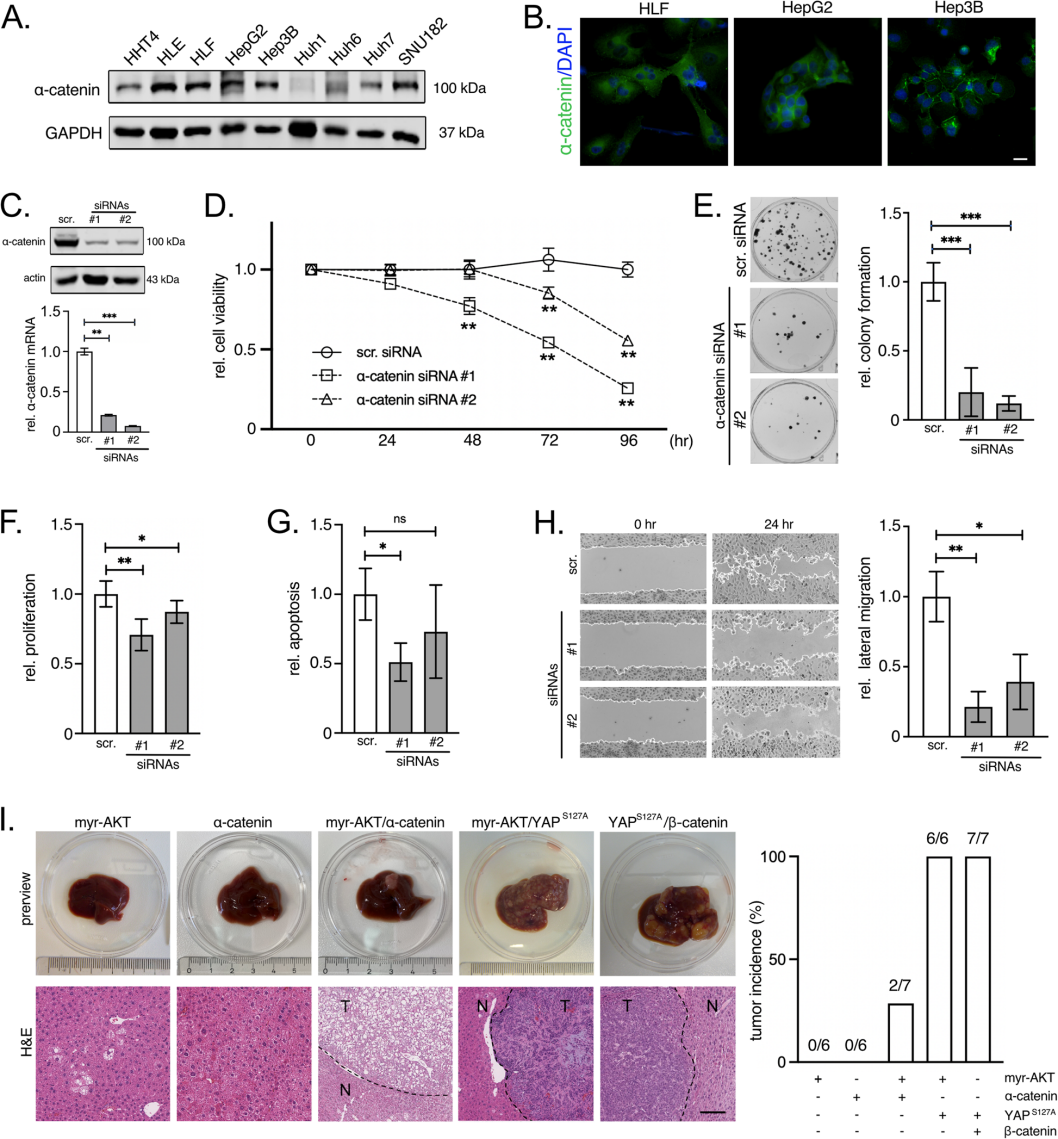
Functional relevance of α‐catenin in hepatocarcinogenesis. **A** Western blot analysis detecting α‐catenin in human liver cancer cell line lysates (*n* = 8) and the immortalized human hepatocyte cell line HHT4. **B** Immunofluorescence images of HLF, HepG2, and Hep3B cells stained for α‐catenin. Nuclei are visualized by DAPI. Scale bar: 10 µm. **C** Representative Western immunoblot and qPCR of α‐catenin in HLF cells after transfection of two independent α-catenin-specific siRNAs (#1, #2). Samples were isolated 72 h after transfection. **D** Cell viability assay of HLF cells after siRNA-mediated silencing of α‐catenin at indicated time points. **E** Colony formation assay using HLF cells after siRNA-mediated α‐catenin knockdown followed by the quantification of relative colony formation (software: ImageJ). Colony density was measured 2 weeks after seeding cells. **F** Proliferation of HLF cells was measured using a BrdU ELISA. Incorporation of the base thymidine analog was detected 96 h after transfection of α‐catenin-specific siRNAs. **G** HLF cell apoptosis was detected utilizing a CellTox™ Green reagent 48 h after inhibition of α‐catenin. **H** Lateral migration of HLF cells was detected at indicated time points using a ‘scratch’ assay after siRNA-mediated α‐catenin inhibition. Cells were pretreated with mitomycin-C to prevent cell mitosis. Relative lateral migration was quantified using ImageJ. **I** Hydrodynamic gene delivery of expression vectors in FVB/N mice. Exemplary pictures of livers for all injected gene combinations are shown (myr-AKT, α‐catenin, and myr-AKT/α‐catenin). The gene combinations myr-AKT/YAP^S127A^ and YAP^S127^/β-catenin lead to the formation of multiple tumors within 12 and 8.5 weeks after injection (positive controls). Representative H&E stains with indicated non-tumor (N) and tumor (T) areas are shown. The bar graph illustrates the number of animals with tumor formation. Scale bar: 60 µm. For all RNAi experiments, scramble (scr.) siRNA-transfected cells served as controls. All results were normalized to respective controls. Statistical test: Mann–Whitney U test, *p ≤ 0.05, **p ≤ 0.01, ***p ≤ 0.001

Because α‐catenin mediates tumor-suppressive properties in other cancer cell types, we asked if α‐catenin overexpression facilitates contrary effects in HCC cells. Indeed, Western immunoblotting illustrated that most liver cancer cells expressed significant amounts of α‐catenin protein (Fig. 2A).

Like in human HCC tissues, immunofluorescence microscopy revealed a variable subcellular localization of α‐catenin: while HLF cells showed a predominant cytoplasmic protein enrichment, HepG2 and Hep3B cells were characterized by a mixed membranous/cytoplasmic localization (with Hep3B having α‐catenin predominantly at the membrane) (Fig. 2B). Additional vector-based expression of α‐catenin in Hep3B cells demonstrated cytoplasmic accumulation of exogenous α‐catenin (data not shown). This spill-over effect after α‐catenin overexpression into the cytoplasm is supported by the fact that only 0.5% of human HCCs showed mutations in the CTNNA1 gene (2/373), excluding gene mutations as possible cause for aberrant cytosolic α‐catenin localization [31].

To further investigate the biological impact of membranous and/or cytoplasmic α‐catenin, silencing of α‐catenin by two independent siRNAs in three cell lines with variable α‐catenin localization was performed (HLF: Fig. 2C, HepG2/Hep3B: Suppl. Figure S2A/B). Indeed, reduction of α‐catenin was associated with significantly diminished cell viability in all investigated cells after 72 and 96 h (up to 75%) (Fig. 2D, Suppl. Figure S2C). The positive effect of α‐catenin on cell proliferation was confirmed by utilizing cell colony formation assays (Fig. 2E, Suppl. Figure S2D) and measurement of cell proliferation by a BrdU ELISA (Fig. 2F, Suppl. Figure S2E). Apoptosis was not consistently affected after α‐catenin silencing in all analyzed cell lines (Fig. 2G, Suppl. Figure S2F). Interestingly, lateral cell migration was strongly diminished after α‐catenin silencing when blocking proliferation with mitomycin-C (Fig. 2H, Suppl. Figure S2G).

Based on the robust tumor-supporting properties in vitro, we hypothesized that α‐catenin may also function as an oncogene in hepatocarcinogenesis. For this, its oncogenic potential was analyzed using hydrodynamic gene delivery, which allows the rapid genetic manipulation of hepatocytes in mice [19]. A vector coding for α‐catenin was injected together with a construct expressing a Sleeping Beauty transposase that facilitates the stable genomic integration of the α‐catenin expression vector exclusively in hepatocytes. A first experiment demonstrated that overexpression of α‐catenin alone did not cause tumor formation up to 12 weeks after injection (Fig. 2I). In contrast, co-injection with the hepatic oncogene AKT (myristoylated-AKT; myr-AKT) led to low frequency tumor formation (few prominent tumor nodules per animal). Indeed, tumor frequency after myr-AKT/α‐catenin expression was drastically lower as compared to injections with constitutively active YAP (YAP^S127A^)/myr-AKT (leading to intrahepatic cholangiocarcinoma) as well as YAP^S127A^/β-catenin (leading to hepatoblastoma) [32, 33]. Histological evaluation revealed that the Ki67-positive tumor nodules initiated by α‐catenin/myr-AKT were positive for the hepatocyte marker HNF4α and showed distinct clusters of the cholangiocytic marker cytokeratin 19 (CK19) (Suppl. Figure S2H). This picture differs from tumors induced by myr-AKT/YAP^S127A^, which stained positive for CK19 in most tumor regions (but were negative for nuclear HNF4α). In contrast, YAP^S127A^/β-catenin-induced hepatoblastoma were characterized by weak CK19 positivity and prominent nuclear HNF4α. Supported by a pathological evaluation, these results illustrated that α‐catenin/myr-AKT-induced tumors showed features of well-differentiated HCCs.

In sum, α‐catenin overexpression supports liver cancer cell proliferation and migration, illustrating its tumor cell-promoting properties in hepatocarcinogenesis. α‐catenin is a weak oncogene, which moderately supports the formation of HCC in conjunction with other oncogenes.

### Defining the interactome of α‐catenin—CEP55 is a new α‐catenin binding partner

**Fig. 3:**
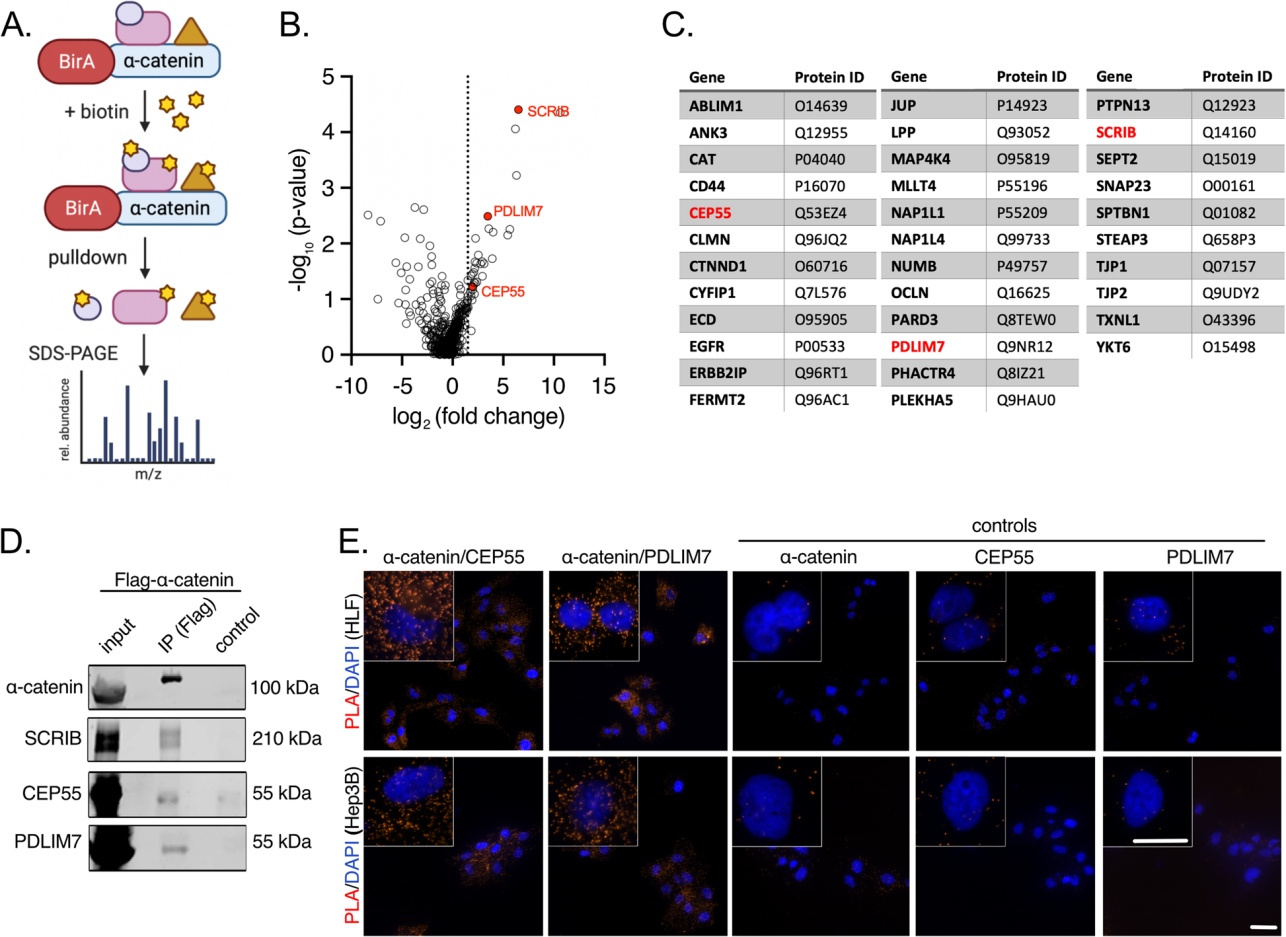
CEP55 and PDLIM7 bind α‐catenin in the cytoplasm of HCC cells. **A** Scheme depicting the experimental setup for the identification of α-catenin binding partners (BioID approach). In presence of biotin, the ligase BirA biotinylates proteins in close proximity to α‐catenin. Biotinylated proteins are affinity purified using streptavidin pulldown and identified by mass spectrometry. Scheme adapted from “BioID Assay”, by BioRender.com (2020). Retrieved from https://app.biorender.com/biorender-templates. **B** Volcano plot illustrating enrichment versus statistical significance of proteins identified by mass spectrometry. The x-axis indicates the log_2_-fold change (FC) and the y-axis the − log_10_ *p*-value. The dashed vertical line represents the cut-off (enrichment > 1.5). Red dots indicate three factors investigated in this study: CEP55, PDLIM7, and SCRIB. **C** List of 34 factors with protein IDs that were identified as potential α-catenin binding partners. Candidates used for further analyses are indicated in red. **D** Co-IP utilizing cell extracts from HLF cells transfected with Flag-tagged human α‐catenin followed by detection of α-catenin, CEP55, or PDLIM7. SCRIB, which is a known interaction partner of the cadherin/catenin complex, was used as positive control. **E** PLA experiments illustrating spatial proximity of α-catenin with CEP55 and PDLIM7 in the cytoplasm of HLF and Hep3B cells. Incubation with antibodies detecting α-catenin, CEP55, or PDLIM7 alone were used as negative controls. Scale bars: 50 and 10 µm for low and high magnifications, respectively

To understand how α‐catenin supports HCC cell proliferation and/or migration in HCC cells, we performed a Bio-ID approach using α‐catenin tagged with the biotin ligase BirA at the N-terminus of the protein (Fig. 3A) [34]. Doxycycline-inducible expression of the fusion protein in combination with biotin led to the biotinylation of several potential interaction partners (Suppl. Figure S3A/B) and subsequent mass spectrometry identified 104 potential α‐catenin binding proteins enriched in HLF cells (Fig. 3B). The 34 most abundant proteins as calculated by intensity-based absolute quantification (iBaq) were used as input for a protein network analysis (Fig. 3C, Suppl. Table 7). Twenty-four of them form a cluster of known interactions at AJ such as afadin (AFDN/MLLT4) [35], plakoglobin (JUP/γ-catenin) [36], or δ-catenin (CTNND1) [37]. In addition, several actin-interacting factors were—to our knowledge—identified for the first time as α‐catenin binding partners, which illustrates the close spatial organization of α‐catenin with the actin cytoskeleton. These factors included calmin (CLMN), limatin (ABLIM1), and PDZ and LIM domain 7 (PDLIM7).

Interestingly, several short-listed proteins that have not been described to interact with α‐catenin point to more direct mechanisms how this junctional factor could control cell viability or migration of tumor cells. Examples were the transcriptional regulator ecdysoneless cell cycle regulator (ECD) or the cytokinesis regulator centrosomal protein 55 (CEP55). For confirmatory experiments, we selected PDLIM7 and CEP55 as well as SCRIB (positive control), the latter being a known binding partner of the cadherin/catenin complex [38].

First, the protein complex formation of α‐catenin/CEP55 and α‐catenin/PDLIM7 was confirmed by independent Co-IP experiments (Fig. 3D). To define the subcellular compartment in which the physical interaction occurred, proximity ligation assay (PLA) analysis with α‐catenin/CEP55 and α‐catenin/PDLIM7 antibody combinations was performed. Indeed, a strong cytoplasmic but not membrane-associated interaction for CEP55 and PDLIM7 with α‐catenin was detectable, confirming our finding that α‐catenin is frequently (mis-) localized in the cytosol of HCC cells (Fig. 3E). Importantly, even for Hep3B with predominant membranous but low cytoplasmic α‐catenin localization (Fig. 2B), spatial proximity between α‐catenin and CEP55 or PDLIM7 was exclusively detectable in the cytoplasm.

Together, we here identified several new α‐catenin interacting partners such as PDLIM7 and CEP55, which exclusively interact with α‐catenin in the cytoplasm of liver cancer cells.

### CEP55 overexpression in HCC cells is regulated by the YAP/TEAD/FoxM1 complex

**Fig. 4:**
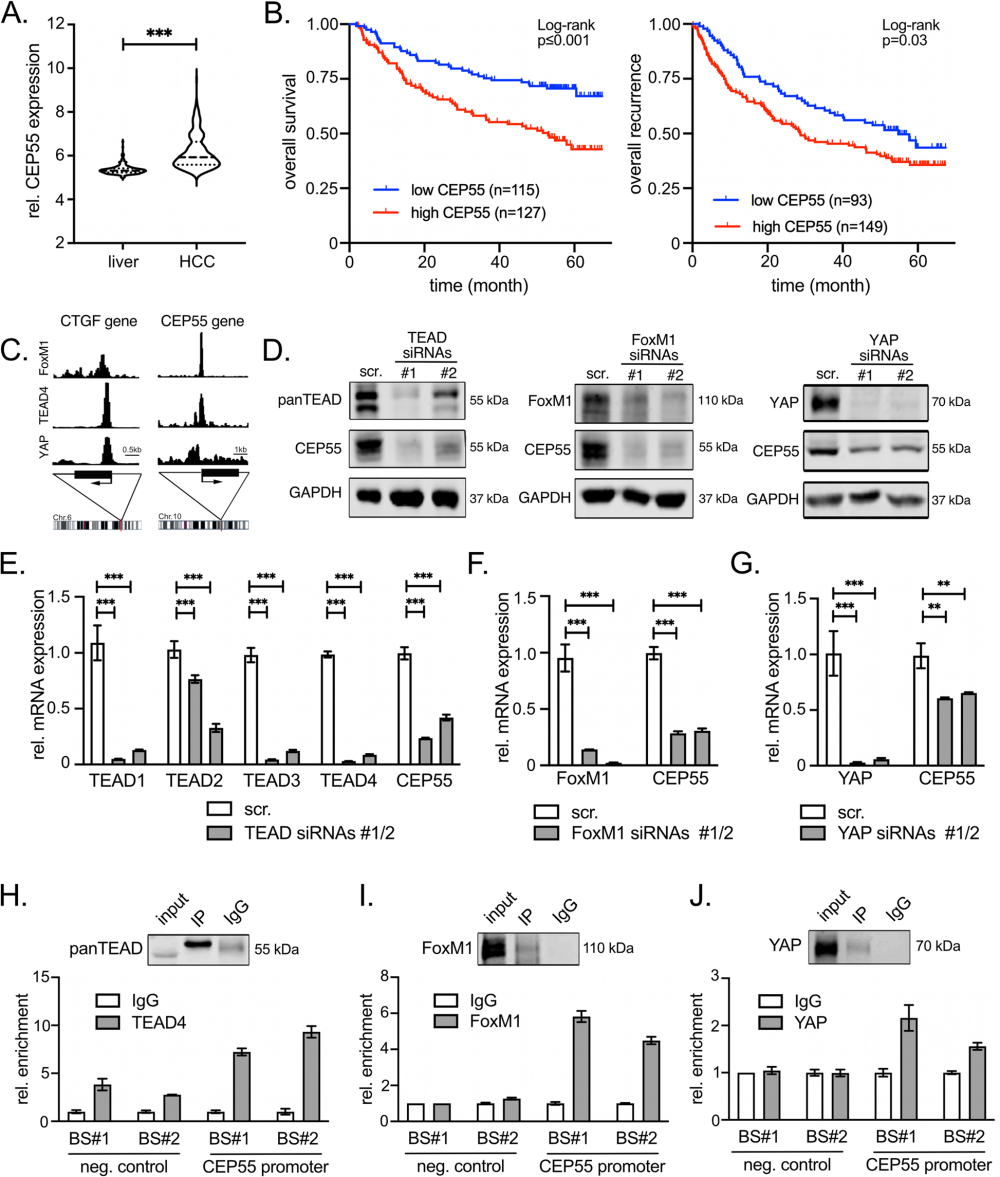
The TEADs/FoxM1/YAP complex regulates CEP55 overexpression in liver cancer cells. **A** CEP55 transcriptome data derived from human HCC tissues and adjacent liver tissues are compared [23]. Statistical test: Mann–Whitney U test. ***p ≤ 0.001. **B** Kaplan–Meier plots of HCC patients showing overall survival and cancer recurrence depending on CEP55 expression. Patients were divided in groups with low and high CEP55 expression using Cutoff Finder. Statistical test: log-rank test. *p*-values and group sizes are indicated. **C** Scheme depicting ChIP-Seq profiles in the promoter region of the human CEP55 gene. Data for FoxM1, TEAD4, and YAP are shown. The TEAD/YAP target gene CTGF is shown as positive control. Binding sites for TEAD4 and FoxM1 in the promoter of CEP55 were selected using the JASPAR database. **D** Western Immunoblot data of HLF cells after gene-specific silencing of TEAD1/3/4 family members, FoxM1, and YAP for 48 h. Due to structural differences, TEAD2 is not efficiently targeted by the chosen siRNAs. **E–G** Real-time PCR analysis of TEAD1-4 and CEP55 (**E**), FoxM1 and CEP55 (**F**), as well as YAP and CEP55 (**G**) after silencing of the respective transcriptional regulator. Analysis was performed using HLF cells. Statistical test: Mann–Whitney U test. **p ≤ 0.01, ***p ≤ 0.001. **H-J** ChIP analysis of TEAD4 (**H**), FoxM1 (**I**), and YAP (**J**) at two predicted binding sites (BS) in the CEP55 promoter (BS#1 and BS#2). Two CEP55 upstream promoter regions served as negative controls (BS#1 and BS#2). IgG was employed as antibody control. Results were normalized to respective IgG controls. Western blots in **H**-**J** illustrate successful IP of TEADs, FoxM1, and YAP

We decided to focus on CEP55 as this protein has been described as regulator of mitosis [39] and also migration [40]. In addition, first data indicated CEP55 overexpression in HCC tissues [41]. Indeed, we confirmed elevated CEP55 levels in different HCC cohorts and showed that CEP55 overexpression correlated with poor patient survival and early cancer recurrence (Fig. 4A/B, Suppl. Figure S4A/B). On the protein level, tissue microarray analyses revealed that CEP55 was detectable in the cytosol of hepatocytes and tumor cells with 38% of all HCC showing a moderate to strong protein enrichment (Suppl. Figure S4C).

In addition, we recognized that CEP55 was part of a gene signature that was characteristic for the presence of chromosomal instability (CIN) in different cancer types (called CIN70) [42]. Because we previously demonstrated that many CIN70 signature genes were cooperatively regulated by the oncogenic transcriptional regulator YAP, TEA domain transcription factors (TEADs), and forkhead box M1 (FoxM1) [42], we hypothesized that CEP55 was controlled by this complex consisting of transcription factors and a co-activator.

First, we investigated if the TEAD family member TEAD4, FoxM1 as well as YAP could physically bind the CEP55 gene promoter. Indeed, the potential binding of TEAD4, FoxM1, and YAP was indicated by published ChIP-Seq data in combination with the JASPAR database (Fig. 4C). Interestingly, predicted sites for direct or indirect DNA interaction were in spatial proximity, supporting previous data showing that a TEAD/FoxM1/YAP complex is required for the regulation of CIN70 genes [42].

The transcriptional regulation of CEP55 by the TEAD/FoxM1/YAP complex was further investigated using RNA*interference* experiments. Inhibition of three TEAD family members (pan-TEAD siRNAs targeting TEAD1, 3, and 4 with moderate effects on TEAD2), FoxM1, or YAP by different siRNAs confirmed a strong CEP55 reduction at the transcript and protein levels (Fig. 4D-G, Suppl. Figure S4D-F). Lastly, we investigated the binding of TEAD4, FoxM1 and YAP at the CEP55 gene promoter using chromatin immunoprecipitation (ChIP) experiments. For all three factors the interaction with two potential binding sites (BS) in the CEP55 gene promoter was demonstrated, while no such binding was detectable outside the promoter (Fig. 4H-J).

In sum, our data demonstrate that overexpression of the α‐catenin binding partner CEP55 is mediated by a transcriptional complex consisting of TEAD/FoxM1/YAP.

### α‐catenin stabilizes CEP55, which controls HCC cell migration

**Fig. 5:**
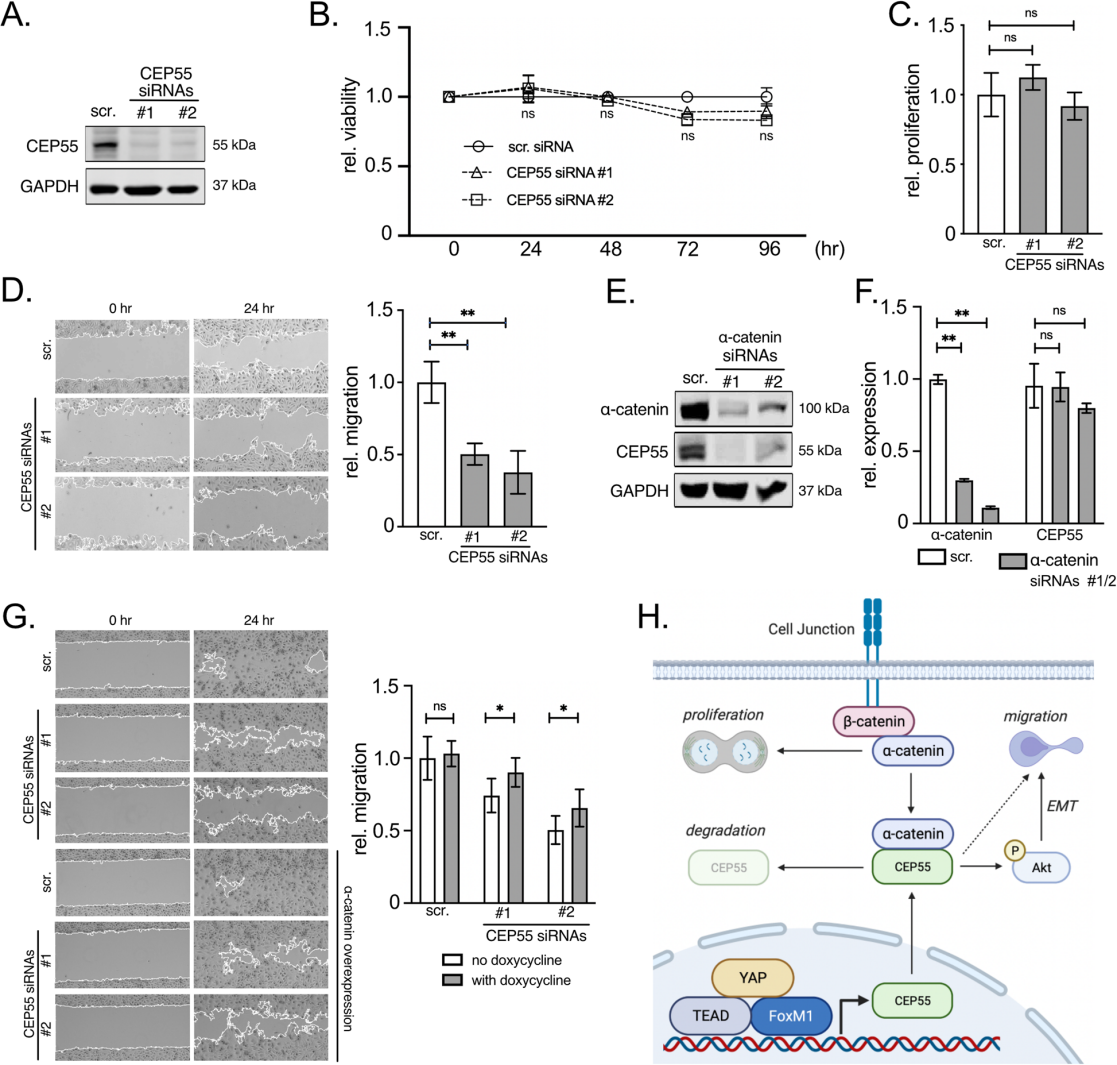
α-catenin stabilizes CEP55 to support HCC cell migration but not proliferation. **A** Western immunoblot of CEP55 in HLF cells after transfection of two gene-specific siRNAs (#1, #2). Samples were isolated 48 h after transfection. **B** Cell viability assay of HLF cells after siRNA-mediated silencing of CEP55 at indicated time points. **C** Proliferation of HLF cells was measured 72 h after siRNA transfection specific for CEP55. **D** Lateral migration of HLF cells after CEP55 silencing was investigated using a ‘scratch’ assay after 24 h. Cells were pretreated with mitomycin-C to block cell proliferation. **E** Western immunoblot after inhibition of α‐catenin by siRNAs in HLF cells. Samples were isolated 72 h after transfection. **F** Real-time PCR analysis of α‐catenin and CEP55 excludes the possibility of transcriptional regulation of CEP55 via an α‐catenin-dependent mechanism. **G** Lateral migration of HLF cells after CEP55 silencing with and without inducible expression of α‐catenin at indicated time points. Representative pictures of scratches are shown. **H** Scheme summarizing findings of this study. While the FoxM1/TEAD/YAP complex is required for the overexpression of CEP55, the interaction between α‐catenin and CEP55 is essential for the stabilization of CEP55. CEP55 facilitates a pro-migratory phenotype, likely via activation of the PI3K/AKT pathway and induction of EMT. Additional molecular mechanisms are possible (dashed arrow). Created with BioRender.com (2020), https://app.biorender.com/biorender-templates. Statistical tests in B., C., D., E., G: Mann–Whitney U test. *p ≤ 0.05, **p ≤ 0.01, ***p ≤ 0.001

We then asked if CEP55 overexpression in HCC cells could contribute to α‐catenin-dependent cell proliferation and/or migration. Interestingly, the efficient silencing of CEP55 by two siRNAs did not significantly affect HCC cell viability or proliferation (Fig. 5A-C, Suppl. Figure S5A/B). These results were surprising as CEP55 was described as an important regulator of mitotic exit and cytokinesis [39]. Instead, CEP55 inhibition significantly reduced lateral cell migration in different HCC cells (Fig. 5D, Suppl. Figure S5C). This finding is consistent with previous work showing that CEP55 (synonym FLJ10540) contributes to cell migration and invasion via activation of the PI3K/AKT pathway [40]. Accordingly, we confirmed that CEP55 positively controls AKT phosphorylation and the expression of genes associated with epithelial-mesenchymal transition (EMT) (Suppl. Figure S5D/E).

Next, we wanted to clarify the mechanistic connection between α‐catenin and CEP55 in liver cancer cells. Indeed, silencing of α‐catenin by RNA*interference* resulted in a strong reduction of CEP55 at the protein level (Fig. 5E). As α‐catenin and CEP55 physically interacted with each other (Fig. 3), these results indicated that α‐catenin was stabilizing CEP55 protein. This assumption was supported by the fact that α‐catenin silencing did not cause significant changes of the CEP55 transcript levels (Fig. 5F).

Based on our results we hypothesized that α‐catenin and CEP55 cooperate in the regulation of HCC migration. This was confirmed by an epistasis experiment illustrating that overexpression of α‐catenin could partly rescue liver cancer cells from effects caused by CEP55 inhibition (Fig. 5G).

In summary, α‐catenin is stabilizing CEP55 protein, which in turn contributes to the pro-migratory effect of α‐catenin in HCC cells.

## Discussion

The formation of proper cell–cell adhesion is essential for cell polarization and spatial organization of tissues and organs. Indeed, alterations in adhesiveness between cells have been recognized several decades ago by pathologists, as normal living epithelial cells adhere stronger to each other than cancer cells [43]. Such observations laid the basis for the hypothesis that aberrant organization of cell junctions is not merely the result of malignant transformation but can itself contribute to tumor formation and cancer progression. Until now, several publications illustrated that junction proteins are critical for cell homeostasis and can contribute to pro-tumorigenic processes and tumor formation via a plethora of molecular mechanisms [44]. However, the mode of action for some cancer-relevant junctional factors such as α‐catenin is not well understood. For example, α‐catenin reduction and tumor-suppressive properties have been described for many tumor types such as colorectal and breast cancer [45, 46]. In contrast, our study and results from other groups demonstrated that α‐catenin is overexpressed in up to 50% of the HCC tissues [16]. In addition, our molecular and functional analyses support the concept that α‐catenin contributes to liver cancer proliferation and migration.

The tumor-supporting effects of α‐catenin are contradictory to previous findings from other cancer types such as bladder cancer (BLCA), where α‐catenin inhibits cancer cell proliferation and invasion [47]. Interestingly, α‐catenin expression is significantly reduced in BLCA (Suppl. Figure S1A), substantiating the hypothesis that α‐catenin induction acts as tumor-promoting event in some cancer entities (e.g., HCC), while in other cancer types α‐catenin reduction supports tumor progression (e.g., BLCA). However, our results also show that α‐catenin overexpression does not represent a strong tumor-initiating event as co-expression with another oncogene is required to cause (rare) tumor formation in vivo. α‐catenin-dependent tumors biochemically and histologically differ from other oncogene-induced tumors as illustrated in our study for myr-AKT/YAP^S127A^ or YAP^S127A^/β-catenin. For example, many tumor cells were negative for CK19 after myr-AKT/α‐catenin injection in mouse livers; however, distinct CK19 positive areas in tumor nodules were also detectable (cholangiocytes in the adjacent liver tissue served as staining control). In intrahepatic cholangiocarcinoma (myr-AKT/YAP^S127A^) a stronger and in hepatoblastoma (YAP^S127A^/β-catenin) a less prominent CK19 positivity in tumor cells was observed [32, 33]. As myr-AKT/α‐catenin overexpression led to a phenotype with features of well-differentiated HCC (CK19 negative and HNF4α positive tumor cells), it is tempting to speculate that α‐catenin moderately supports liver cancer formation and tumor differentiation via distinct and β-catenin-independent mechanisms. Future studies must clarify how α‐catenin mechanistically affects malignant transformation in conjunction with activated AKT or other liver oncogenes.

Next to the elevated expression of α‐catenin, our study also demonstrates a cytoplasmic localization of α‐catenin in HCC tissues and cultured tumor cells. Indeed, the ‘unexpected’ cytoplasmic and even nuclear localization of factors from cell junctions and polarity complexes has been demonstrated previously. For example, the AJ protein E-cadherin can translocate with β-catenin to the nuclear compartment of colorectal cancer cells [48]. Equally, the basolateral cell polarity complex protein Scribble (SCRIB) supports liver cancer initiation and migration upon enrichment in the cytosol [18]. Our results for the cytoplasmic accumulation of α‐catenin in HCC cells is supported by previous studies demonstrating a similar cytosolic phenotype for other tumor types such as squamous cell carcinoma, thyroid neoplasm, and breast cancer [49–51]. Although the molecular mechanisms behind this observation are not known, our results suggest a mechanism how cytoplasmic α‐catenin could contribute to tumor progression. Because we observed an exclusive cytoplasmic and no membranous interaction between α‐catenin and CEP55 (even in Hep3B cells with clear membranous α‐catenin, Figs. 2B and 3E), it is tempting to speculate that α‐catenin overexpression is leading to its cytosolic enrichment and subsequent interaction with other proteins in this cellular compartment. Thus, aberrant α‐catenin localization upon elevated expression could stabilize a panel of proteins with individual cancer-supporting effects.

Importantly, the cytoplasmic stabilization of CEP55 by α‐catenin is just one possibility of how CEP55 expression is regulated in tumor cells. CEP55 is part of the CIN70 gene signature that is indicative of CIN and previous data from our group demonstrated that many CIN signature genes are transcriptionally regulated by YAP, TEADs, and FoxM1 [42]. Because we also experimentally confirmed the transcriptional regulation of CEP55 by this complex, several molecular processes contribute to the enrichment of CEP55 in HCC cells (protein stabilization and transcriptional regulation; Fig. 5H). Indeed, CEP55 has been described to be aberrantly expressed in many cancers, such as breast cancer and HCC, where its overexpression was considered to promote CIN [52, 53]. However, CEP55 did not show pro-proliferative properties in our cell systems, even though it has been recognized as a centrosomal protein required for mitotic exit and cytokinesis [39]. Currently, there is no mechanistic explanation for the lack of a pro-proliferative phenotype in HCC cells; however, one possible scenario is that other proteins functionally compensate for the loss of CEP55 in case of its genetic silencing in HCC cells. It is interesting to note that α‐catenin, which stabilizes CEP55, positively controls both proliferation and migration. Thus, α‐catenin facilitates its migration-supporting effects partly via CEP55, while other molecular processes/proteins/interaction partners be responsible for the pro-proliferative phenotype of α‐catenin (Fig. 5H).

Our results suggest that CEP55 controls the migratory phenotype of HCC cells via regulation of the AKT signaling pathway. This connection was previously confirmed by several studies in different cancer cell types [40, 54]. Indeed, published data suggests how CEP55 may contribute to a pro-migratory and pro-invasive phenotype. For example, CEP55 is a microtubule (MT)-associated protein, which controls MT bundling and probably MT dynamics [55]. As dynamics of this cytoskeletal network is crucial for cell division but also migration, it is possible that CEP55 overexpression in HCC cells affects cell mobility via the regulation of MT polymerization. Indeed, evidence demonstrates that MT-interacting proteins that control MT catastrophe or elongation participate in cell movement as illustrated for MAP2 and stathmin [56, 57].

Are there therapeutic implications of our study? Previous studies already identified CEP55 as potential target for anticancer therapies. Although, no specific small molecule inhibitors against CEP55 exist to our knowledge, first computational approaches for the design of future drugs have been performed [58]. However, alternative approaches targeting upstream regulatory mechanisms of CEP55 may also present possible strategies to block its tumor-supporting properties. For example, MEK1/2 inhibition was proposed as one possible perturbation approach as the MAPK pathway transcriptionally controls CEP55 expression in breast cancer cells [52]. In addition, pharmacological inactivation of the YAP/TEAD complex may represent an attractive opportunity as first drugs targeting this transcriptional complex are currently investigated and can be expected soon [59, 60].

Together, our data demonstrate that overexpression of the AJ constituent α‐catenin supports tumor initiation, proliferation, and migration of HCC cells. Both cytosolic α‐catenin-dependent CEP55 stabilization and YAP/TEAD/FoxM1-dependent transcriptional CEP55 induction contribute to the enrichment of pro-migratory CEP55.

## Conclusions

The loss of cell–cell contact between epithelial cells is not merely a secondary effect in the process of human carcinogenesis. Instead, the dysregulation of individual junction proteins contributes to tumor formation and progression via distinct oncogenic mechanisms. We demonstrate that overexpression of α‐catenin contributes to liver cancer cell migration through physical binding and protein stabilization of CEP55. As CEP55 is also transcriptionally regulated by the YAP/TEAD/FoxM1 complex, several molecular mechanisms cooperate in CEP55-dependent tumor cell dissemination.

## Supplementary Information

**Additional file 1: Suppl. Table 1.** Solutions and Buffers. **Suppl. Table 2.** Cloning primers. **Suppl. Table 3.** siRNA sequences. **Suppl. Table 4.** Primers used for qPCR. **Suppl. Table 5.** Antibodies. **Suppl. Table 6.** Primers for ChIP.

**Additional file 2: Figure S1.** Expression of α‐catenin mRNA in HCC patient cohorts.Comparison of α‐catenin transcriptome data derived from the TCGA database. Twenty-three tumor types for which tumorous and non-malignant tissue data exist were investigated. Nine tumor entities for which no statistical differences were observed are not shown. Urothelial bladder carcinoma, colorectal cancer, kidney chromophobe, kidney renal clear cell carcinoma, lung squamous cell carcinoma, pheochromocytoma and paraganglioma, breast cancer, cervical squamous cell carcinoma, cholangiocarcinoma, kidney renal papillary cell carcinoma, liver hepatocellular carcinoma, stomach adenocarcinoma, thyroid cancer, uterine corpus endometrial carcinoma. Statistical test: Mann-Whitney U test. **p≤0.01; ***p≤0.001.α‐catenin expression analysis of TCGA HCC patient cohort [29]. The cohort includes 363 HCCs and 50 normal liver tissues. Statistical test: Mann-Whitney U test. ***p≤0.001.Kaplan-Meier plots showing HCC patient survival and tumor recurrence depending on α-catenin mRNA expression [29]. Patients were divided in two groups with low and high α-catenin expression using Cutoff Finder. Statistical test: Log-rank test. *p*-values and group sizes are indicated.Bar graph summarizing the distribution of membranous α‐catenin positivity in normal livers, DNs, and HCCs. Statistical test: Mann-Whitney U test. $: normal livers were used for statistical comparison. **p≤0.01; ***p≤0.001.

**Additional file 3: Figure S2.** Functional relevance of α‐catenin in hepatocarcinogenesis.Representative Western immunoblot for α‐catenin in HepG2 and Hep3B cells after transfection of two α‐catenin-specific siRNAs. Samples were isolated 72 h after transfection.qPCR results for α‐catenin in HepG2 and Hep3B cells after transfection of α‐catenin-specific siRNAs. Samples were isolated 72 h after transfection.Cell viability assays of HepG2 and Hep3B cells after siRNA-mediated silencing of α‐catenin at indicated time points.Colony formation assay with Hep3B cells after siRNA-mediated α‐catenin knockdown. Colony density was measured 2 weeks after cell seeding. HepG2 cells were not analyzed due to their inability to form sufficient colonies.Proliferation of HepG2 and Hep3B cells was measured using a BrdU ELISA 96 h after α‐catenin-specific siRNA transfection.HepG2 and Hep3B cell apoptosis was detected 48 h after inhibition of α‐catenin.Lateral migration of Hep3B cells was detected after siRNA-mediated α‐catenin silencing immediately after 'scratching' and after 24 h. To avoid proliferation effects on migration measurement, cells were pretreated with mitomycin-C. HepG2 cell were not used for this assay due to their limited migratory capacity.Hydrodynamic gene delivery of myr-AKT, α‐catenin, myr-AKT/α‐catenin, myr-AKT/YAP^S127A^, and YAP^S127^/β-catenin. Exemplary pictures for the proliferation marker Ki67, the hepatocyte marker HNF4α, and the cholangiocyte marker CK19 are shown. The gene combinations myr-AKT/YAP^S127A^ and YAP^S127^/β-catenin were used as controls. Non-tumorand tumorareas are shown. Scale bar: 60 µm. For all RNAi experiments, scramblesiRNA-transfected cells served as controls. All results were normalized to respective controls. Statistical test: Mann-Whitney U test, *p≤0.05; **p≤0.01, ***p≤0.001.

**Additional file 4: Figure S3.** Identification of α-catenin binding partners using the BioID assay.Western immunoblot analysis illustrates the inducible expression of BirA-tagged α-catenin in HLF cells. A vector expressing only BirA was used as negative control.Combined treatment of cells with doxycycline and biotin for 24 h leads to biotinylation/laddering of proteins in cells expressing BirA or BirA-α-catenin. The intensity of laddering is weaker in cells with BirA-α-catenin expression compared to BirA expression alone, which is indicative for the specificity of the approach.

**Additional file 5: Suppl. Table 7.** Potential α‐catenin binding partners.

**Additional file 6: Figure S4.** The TEADs/FoxM1/YAP complex regulates CEP55 in liver cancer cells.CEP55 expression analysis in an independent HCC patient cohort. Statistical test: Mann-Whitney U test. ***p≤0.001.Kaplan-Meier plots of overall patient survival and cancer recurrence according to low and high CEP55 expression [29]. Patients were divided in groups using Cutoff Finder. Statistical test: Log-rank test. *p*-values and group sizes are indicated.Representative images of immunohistochemistry stains for CEP55 with low and high expression. The pie chart illustrates the percentage of different CEP55 expression levels in the subgroup of HCC. The HCC tissue microarray contains normal livers, DNsand HCCs. Scale bars: 60 µm.Real-time PCR analysis of TEAD1-4 and CEP55, FoxM1 and CEP55, as well as YAP and CEP55after silencing of the respective transcriptional regulators by two siRNAsin HLE cells. For all RNAi experiments, scramblesiRNA-transfected cells served as controls. Statistical test: Mann-Whitney U test. *p≤0.05, **p≤0.01, ***p≤0.001.

**Additional file 7: Figure S5.** CEP55 supports HCC cell migration but not proliferation.Western immunoblot for CEP55 in Hep3B cells after transfection of two gene-specific siRNAs. Samples were isolated 48 h after transfection.Cell viability assay and proliferation assay of Hep3B cells after siRNA-mediated silencing of CEP55. Viability was measured at indicated timepoints and proliferation was detected after 72 h.Lateral migration of Hep3B cells after CEP55 silencing was detected using a 'scratch' assay after 24 h. Cells were pretreated with mitomycin-C.Western immunoblot after CEP55 silencing in HLF and Hep3B cells. The expression of AKT, its phosphorylation, and the expression of the EMT-related protein Snai1were analyzed.Real-time PCR analysis of the EMT genes Snai1 and ZEB1 after RNAi-mediated CEP55 inhibition. Statistical test used in,, and: Mann-Whitney U test. **p≤0.01, ***p≤0.001.

**Additional file 8. **

## Data Availability

The datasets generated and/or analyzed during the current study are available at the University of Alabama at Birmingham Cancer data analysis portal (UALCAN) [22]. In addition, a previously published independent HCC cohort was analyzed [23]. TEAD4 and FoxM1 ChIP-Seq data from HepG2 cells were obtained from the ENCODE project (GSE170161 and GSE169998, respectively) [24]. YAP ChIP-Seq data from NCI-H2052 cells were retrieved from GEO (GSE61852) [25]. The mass spectrometry proteomics data have been deposited to the ProteomeXchange Consortium via the PRIDE partner repository (https://www.ebi.ac.uk/pride/; PXD039222).

## Abbreviations

AJ: Adherens junction
CEP55: Centrosomal protein 55
EMT: Epithelial-mesenchymal transition
FoxM1: Forkhead box M1
HCC: Hepatocellular carcinoma
TEAD: TEA domain transcription factor
YAP: Yes-associated protein
