# Supplementary material for: α-catenin interaction with YAP/FoxM1/TEAD-induced CEP55 supports liver cancer cell migration
Source: Cell Commun Signal. 2023 Jun 28;21:162. doi: 10.1186/s12964-023-01169-2 (PMC10304383; doi:10.1186/s12964-023-01169-2)
Supplement: Supplementary file 2 — Additional file 1: Suppl. Table 1. Solutions and Buffers. Suppl. Table 2. Cloning primers. Suppl. Table 3. siRNA sequences. Suppl. Table 4. Primers used for qPCR. Suppl. Table 5. Antibodies. Suppl. Table 6. Primers for ChIP. [file 12964_2023_1169_MOESM1_ESM.docx]

**α-catenin interaction with YAP/FoxM1/TEAD-induced CEP55 supports liver cancer cell migration**

Y. Tang, L. Thiess, S.M.E. Weiler, M. Tóth, F. Rose, S. Merker, T. Ruppert, P. Schirmacher, K. Breuhahn

**Suppl. Table 1: Solutions and Buffers**

| Buffer/Solution | Ingredients |
| --- | --- |
| BioID lysis buffer | 50 mM Tris pH 7,5, 200 mM NaCl, 0,1% SDS, 1% Triton X-100, 1 mM EDTA, 0,25% Na Desoxychelat |
| Blocking solution | 5% BSA in TBST |
| Borate buffer (pH 8.8) | 20 mM boric acid, 1.27 mM EDTA |
| Co-IP lysis buffer | 50 mM tris-HCl pH 7,4, 1% NP-40, 0,25 % Na-deoxycholate, 150 mM NaCl, 1 mM EDTA |
| IP wash buffer | 100mM tris pH 8.5, 500 mM LiCl, 1% NP40, 1% Na-desoxycholic acid |
| Laemmli buffer | 65.8 mM tris-HCl pH 6.8, 2.1% SDS, 26.3% (w/v) glycerol, 0.01% bromophenol blue |
| PBS (pH 7.4) | 140 mM NaCl, 2.7 mM KCl, 10 mM Na_2_HPO_4_x2H_2_O, 1.8 mM KH_2_PO_4_ |
| PBST (pH 7.4) | PBS + 0.02% Tween 20 |
| RIPA buffer | 150 mM NaCl, 0.1% SDS, 0.5% Na-deoxycholate, 1% Igepal CA 630, 5 mM EDTA, 50 mM tris pH 8, filter sterile |
| SDS running buffer | 25 mM tris, 192 mM glycin, 0.1% SDS |
| TAE buffer (pH 8) | 40 mM tris acetate, 1 mM EDTA |
| Taliniadis elution buffer | 70 mM tris pH 8, 1 mM EDTA, 1.5% SDS |
| TBST | 0.1% Tween 20 in TBS |
| TE buffer | 70 mM tris pH 8, 1 mM EDTA |
| Tris buffered saline (TBS, pH 7.6) | 20 mM tris-HCl, 140 mM NaCl |

**Suppl. Table 2: Cloning primers**

| Primers for cloning (h/m) | Comment | Sequence (5'-3') |
| --- | --- | --- |
| CTNNA1 | Amplification of hCTNNA1 with attB1 (forward) and attB2 (reverse) recombination sites | for: GGGACAAGTTTGTACAAAAAAGCAGGCTATACC ATGACTGCTGTCCATGCAGG |
|  |  | rev: GGGGACCACTTTGTACAAGAAAGCTGGGTT TTAGATGCTGTCCATAGCTTTG |
| CTNNA1 | Amplification of hCTNNA1 with BgIII (forward) and EcoRI (reverse) recombination sites | for: GATCAGATCTATGACTGCTGTCCATGCAGG |
|  |  | rev: TTCAAAGCTATGGACAGCATCTAAGAATTCGCTC |

### **Suppl. Table 3:** **siRNA sequences**

| siRNA | GenBank ID | Sequence (5'-3') |
| --- | --- | --- |
| Scramble | Eurofins Genomics GmbH | UGGUUUACAUGUCGACUAA-dT-dT |
| AKT #1 | NM_001382431 | GACAGAGGAGCAAGGUUUATT-dT-dT |
| AKT #2 | NM_001243027 | AACAACUUCUCCGUAGCAGAATT-dT-dT |
| CEP55 #1 | NM_001127182 | GCGGGAAGUCUAUGUAAAA-dT-dT |
| CEP55 #2 | NM_001127182 | GCAUCAAUUGCAUGUAAUU-dT-dT |
| CTNNA1 #1 | NM_001290307 | CCUCAGAGAUGGACAACUA-dT-dT |
| CTNNA1 #2 | NM_001290307 | AAGCGAAGAUUGCGGAACA-dT-dT |
| FoxM1 #1 | NM_001243088 | AUAUUCACAGCAUCAUCAC-dT-dT |
| FoxM1 #2 | NM_001243088 | GGACCACUUUCCCUACUUU-dT-dT |
| pan-TEAD #1 | NM_021961; NM_001395214; NM_201443 | AUGAUCAACUUCAUCCACATT-dT-dT |
| pan-TEAD #2 | NM_021961; NM_001395214; NM_201443 | UCAACUUCAUCCACAACCUTT-dT-dT |
| YAP #1 | NM_001282099 | CCACCAAGCUAGAUAAAGA-dT-dT |
| YAP #2 | NM_001282099 | GGUCAGAGAUACUUCUUAA-dT-dT |

**Suppl. Table 4: Primers used for qPCR**

| Primers for qPCR | GenBank ID | Sequence (5'-3') |
| --- | --- | --- |
| B2M | NM_004048.4 | for: CACGTCATCCAGCAGAGAAT |
|  |  | rev: TGCTGCTTACATGTCTCGAT |
| GAPDH | NM_001256799.3 | for: CTGGTAAAGTGGATATTGTTGCCAT |
|  |  | rev: TGGAATCATATTGGAACATGTAAACC |
| CEP55 | NM_001127182.2 | for: AGTAAGTGGGGATCGAAGCCT |
|  |  | rev: CTCAAGGACTCGAATTTTCTCCA |
| CTNNA1 | NM_001290307.3 | for: CGGACTGGAGGGAGACAAAG |
|  |  | rev: AGCAGTCATTTCTGCGGCT |
| FoxM1 | NM_001243088.2 | for: ATAGCAAGCGAGTCCGCATT |
|  |  | rev: TTCCTCCCCAGGCTGGATTT |
| YAP | NM_001282099.2 | for: CCTGCGTAGCCAGTTACCAA |
|  |  | rev: CCATCTCATCCACACTGTTC |
| TEAD1 | NM_021961.6 | for: GACAGTCACCTGTTCCACCAAAG |
|  |  | rev: CCATTCTCAAACCTTGCATACTCCG |
| TEAD2 | NM_001256659.2 | for: CTCACCTGTTCCTCCAAGGTC |
|  |  | rev: CACCAGGTACTCGCACATGG |
| TEAD3 | NM_001395214.1 | for: TTCATGGAGGTGCAGCGAGAC |
|  |  | rev: CGCACATCTACTGCCTCCAG |
| TEAD4 | NM_201443.3 | for: TGGAGTTCTCTGCCTTCCTG |
|  |  | rev: GGACTGGCCAATGTGCACGA |

**Suppl. Table 5: Antibodies**

| Primary Antibody (clone) | Dilution | Source/Company | Cat. No; Identifier |
| --- | --- | --- | --- |
| β-Actin (13E5) | WB: 1:1,000 | Cell Signaling | #4970; RRID: AB_2223172 |
| AKT | WB: 1:1,000 | Cell Signaling | #9272; RRID: AB_329827 |
| pAKT (D9E) | WB: 1:1,000 | Cell Signaling | #4060; RRID: AB_2315049 |
| Biotin | WB: 1:1,000 | DAKO | E0354; RRID: AB_2687571 |
| CEP55 (D1L4H) | WB: 1:1,000  IF (PLA): 1:200 | Cell Signaling | #81693; RRID: AB_2799982 |
| CEP55 (EPR11944) | IHC: 1:200 | Abcam | ab170414; RRID:AB_2665499 |
| CK19 (EPNCIR127B) | IHC: 1:100 | Abcam | ab133496; RRID: AB_11155282 |
| CTNNA1 (G-11) | WB: 1:400  IF (PLA): 1:100 | Santa Cruz (Dallas, USA) | sc-9988; RRID: AB_626805 |
| DYKDDDDK (D6W5B) | WB: 1:1,000 | Cell Signaling | #14793; RRID: AB_2572291 |
| Flag (M2) | IP: 2 μg | Sigma Aldrich | F1804; RRID: AB_262044 |
| FoxM1 (D12D5) | WB: 1:1,000 | Cell Signaling | #5436; RRID: AB_10692483 |
| FoxM1 (G-5) | ChIP: 2 μg | Santa Cruz | sc-376471; RRID: AB_11150135 |
| GAPDH (14C10) | WB: 1:1,000 | Cell Signaling | #2118; RRID: AB_561053 |
| GFP (D5.1) | IHC: 1:100 | Cell Signaling | #2912; RRID: AB_1281300 |
| HNF4a (C11F12) | IHC: 1:100 | Cell Signaling | #3113; RRID: AB_2295208 |
| Ki67 | IHC: 1:500 | Abcam | Ab15580; RRID: AB_443209 |
| Pan-TEAD (D3F7L) | WB: 1:1,000 | Cell Signaling | #13295; RRID: AB_2687902 |
| PDLIM7 | WB: 1:3,000  IF (PLA): 1:100 | Proteintech  (Planegg, Germany) | 10221-1-AP; RRID: AB_2161789 |
| YAP (D8H1X) | WB: 1:400  ChIP: 2 μg | Cell Signaling | #14074; RRID: AB_2650491 |
| YAP | WB: 1:400 | Cell Signaling | #4912; RRID: AB_2218911 |

ChIP: Chromatin immunoprecipitation, IP: immunoprecipitation, IF (PLA): Immunofluorescence, IHC: Immunohistochemistry, WB: Western Blotting

| Secondary Antibody | Source/Company | Cat. No; Identifier |
| --- | --- | --- |
| IRDye 680LT anti-mouse IgG | LI-COR Biosciences | P/N 925-68022; RRID: AB_2814906 |
| IRDye 680LT anti-rabbit IgG | LI-COR Biosciences | P/N 925-68023; RRID: AB_2814907 |
| IRDye 800CW anti-mouse IgG | LI-COR Biosciences | P/N 925-32212; RRID: AB_2716622 |
| IRDye 800CW anti-rabbit IgG | LI-COR Biosciences | P/N 925-32213; RRID: AB_2715510 |

**Suppl. Table 6: Primers for ChIP**

| ChIP primers (h) | Ensembl ID | Sequence (5'-3') |
| --- | --- | --- |
| CTGF Promoter Primers |  | Cell Signaling (#14927) |
| CTGF Upstream Primers |  | Cell Signaling (#14928) |
| CEP55 promoter 1 | ENSG00000138180 | for: CAGTGACGTGCCCCGC |
|  |  | rev: GCCGAGTCACACCATCAGG |
| CEP55 promoter 2 | ENSG00000138180 | for: GACGTGCCCCGCCCT |
|  |  | rev: GGCCGAGTCACACCATCA |
| CEP55 control 1 | ENSG00000138180 | for: CCATACCCTGCTGGTTTGGATA |
|  |  | rev: AGGCTTCGATCCCCACTTA |
| CEP55 control 2 | ENSG00000138180 | for: TGGGGATCGAAGCCTAGTAAC |
|  |  | rev: CCACTGATGTCTTTAAGTGTGCAAT |
