# Supplementary figures and images for: α-catenin interaction with YAP/FoxM1/TEAD-induced CEP55 supports liver cancer cell migration
Source: Cell Commun Signal. 2023 Jun 28;21:162. doi: 10.1186/s12964-023-01169-2 (PMC10304383; doi:10.1186/s12964-023-01169-2)

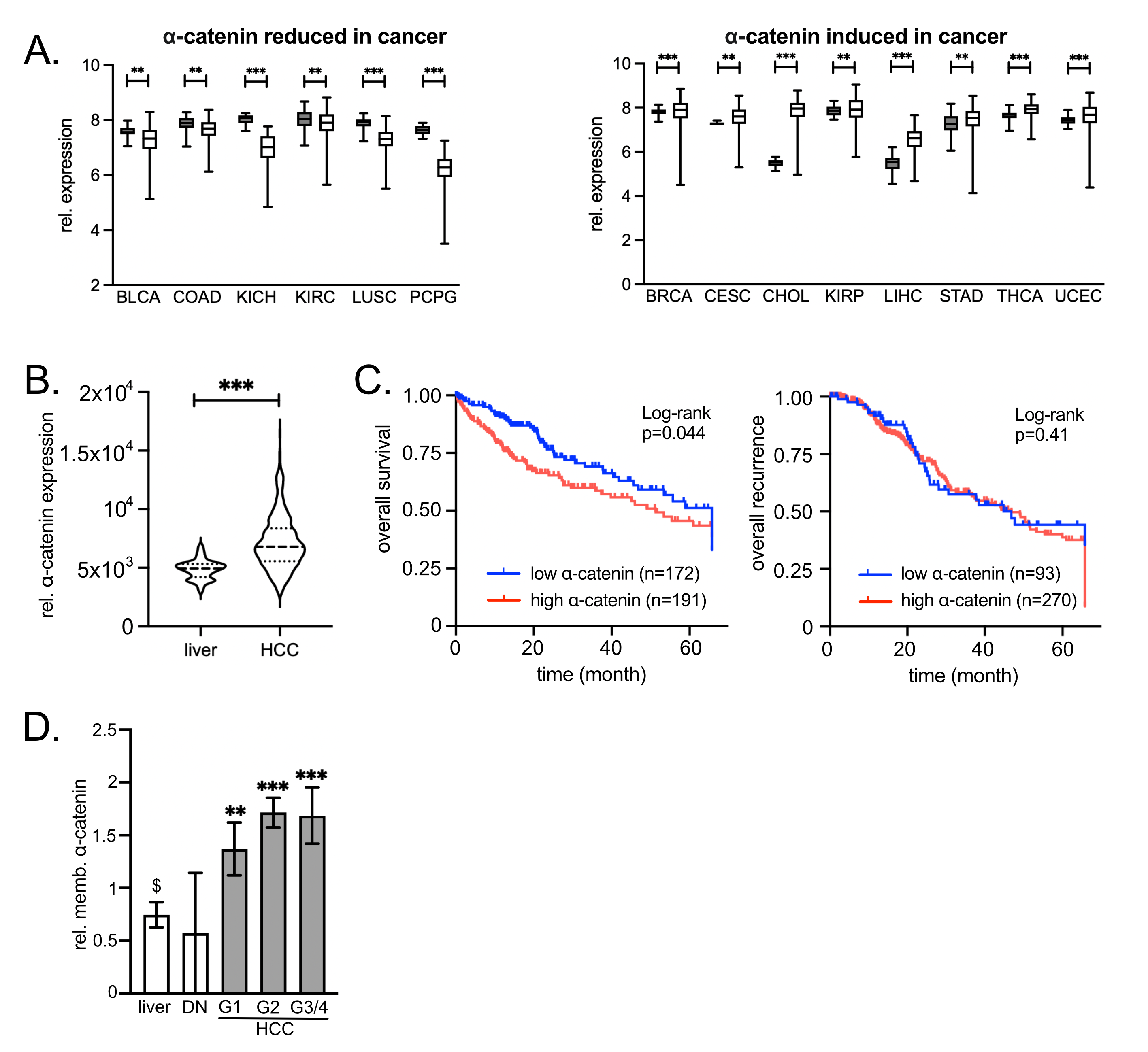

Supplement: Supplementary file 3 — Additional file 2: Figure S1. Expression of α‐catenin mRNA in HCC patient cohorts.Comparison of α‐catenin transcriptome data derived from the TCGA database. Twenty-three tumor types for which tumorous and non-malignant tissue data exist were investigated. Nine tumor entities for which no statistical differences were observed are not shown. Urothelial bladder carcinoma, colorectal cancer, kidney chromophobe, kidney renal clear cell carcinoma, lung squamous cell carcinoma, pheochromocytoma and paraganglioma, breast cancer, cervical squamous cell carcinoma, cholangiocarcinoma, kidney renal papillary cell carcinoma, liver hepatocellular carcinoma, stomach adenocarcinoma, thyroid cancer, uterine corpus endometrial carcinoma. Statistical test: Mann-Whitney U test. **p≤0.01; ***p≤0.001.α‐catenin expression analysis of TCGA HCC patient cohort [29]. The cohort includes 363 HCCs and 50 normal liver tissues. Statistical test: Mann-Whitney U test. ***p≤0.001.Kaplan-Meier plots showing HCC patient survival and tumor recurrence depending on α-catenin mRNA expression [29]. Patients were divided in two groups with low and high α-catenin expression using Cutoff Finder. Statistical test: Log-rank test. p-values and group sizes are indicated.Bar graph summarizing the distribution of membranous α‐catenin positivity in normal livers, DNs, and HCCs. Statistical test: Mann-Whitney U test. $: normal livers were used for statistical comparison. **p≤0.01; ***p≤0.001. [file 12964_2023_1169_MOESM2_ESM.tiff]

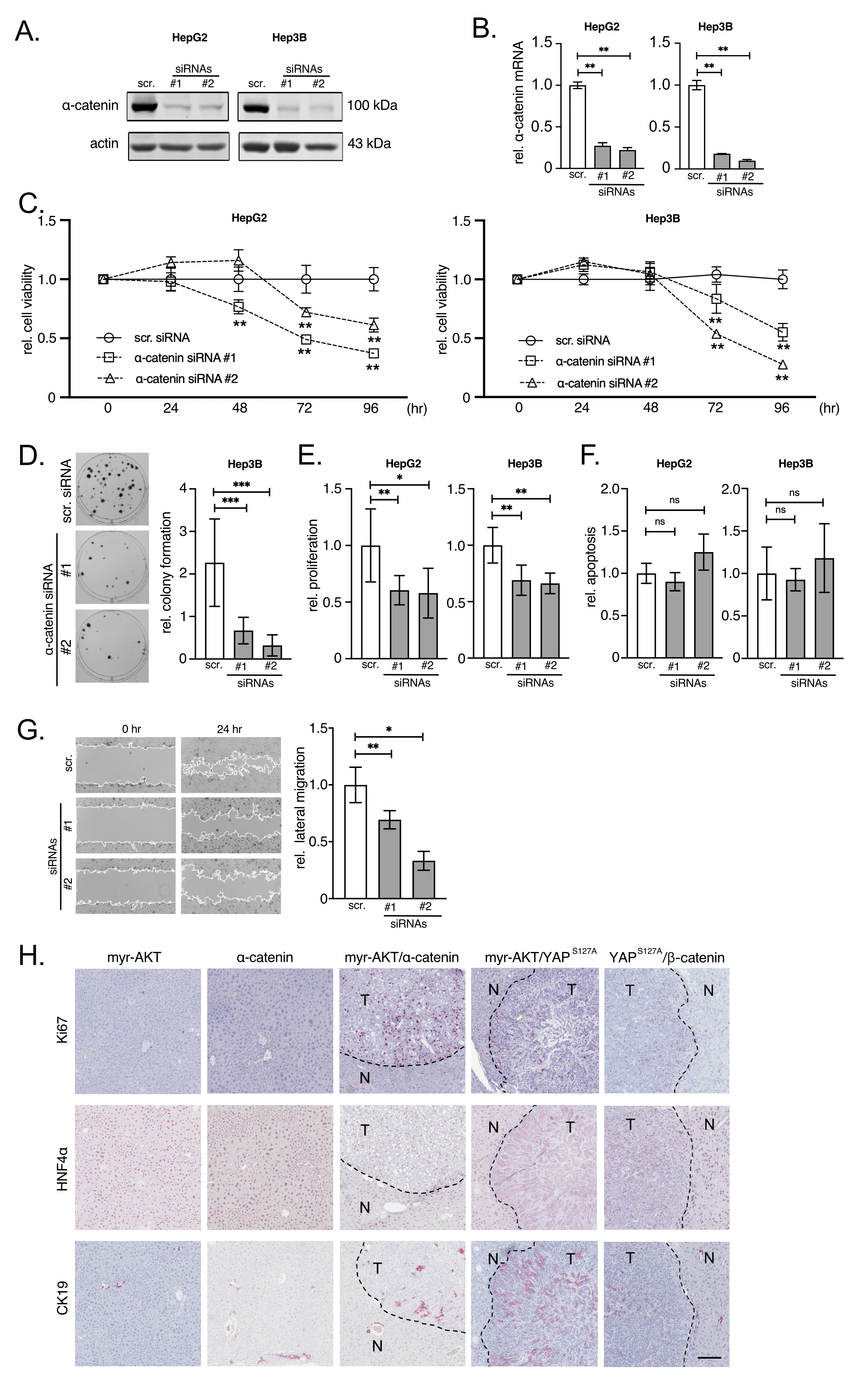

Supplement: Supplementary file 4 — Additional file 3: Figure S2. Functional relevance of α‐catenin in hepatocarcinogenesis.Representative Western immunoblot for α‐catenin in HepG2 and Hep3B cells after transfection of two α‐catenin-specific siRNAs. Samples were isolated 72 h after transfection.qPCR results for α‐catenin in HepG2 and Hep3B cells after transfection of α‐catenin-specific siRNAs. Samples were isolated 72 h after transfection.Cell viability assays of HepG2 and Hep3B cells after siRNA-mediated silencing of α‐catenin at indicated time points.Colony formation assay with Hep3B cells after siRNA-mediated α‐catenin knockdown. Colony density was measured 2 weeks after cell seeding. HepG2 cells were not analyzed due to their inability to form sufficient colonies.Proliferation of HepG2 and Hep3B cells was measured using a BrdU ELISA 96 h after α‐catenin-specific siRNA transfection.HepG2 and Hep3B cell apoptosis was detected 48 h after inhibition of α‐catenin.Lateral migration of Hep3B cells was detected after siRNA-mediated α‐catenin silencing immediately after 'scratching' and after 24 h. To avoid proliferation effects on migration measurement, cells were pretreated with mitomycin-C. HepG2 cell were not used for this assay due to their limited migratory capacity.Hydrodynamic gene delivery of myr-AKT, α‐catenin, myr-AKT/α‐catenin, myr-AKT/YAPS127A, and YAPS127/β-catenin. Exemplary pictures for the proliferation marker Ki67, the hepatocyte marker HNF4α, and the cholangiocyte marker CK19 are shown. The gene combinations myr-AKT/YAPS127A and YAPS127/β-catenin were used as controls. Non-tumorand tumorareas are shown. Scale bar: 60 µm. For all RNAi experiments, scramblesiRNA-transfected cells served as controls. All results were normalized to respective controls. Statistical test: Mann-Whitney U test, *p≤0.05; **p≤0.01, ***p≤0.001. [file 12964_2023_1169_MOESM3_ESM.tiff]

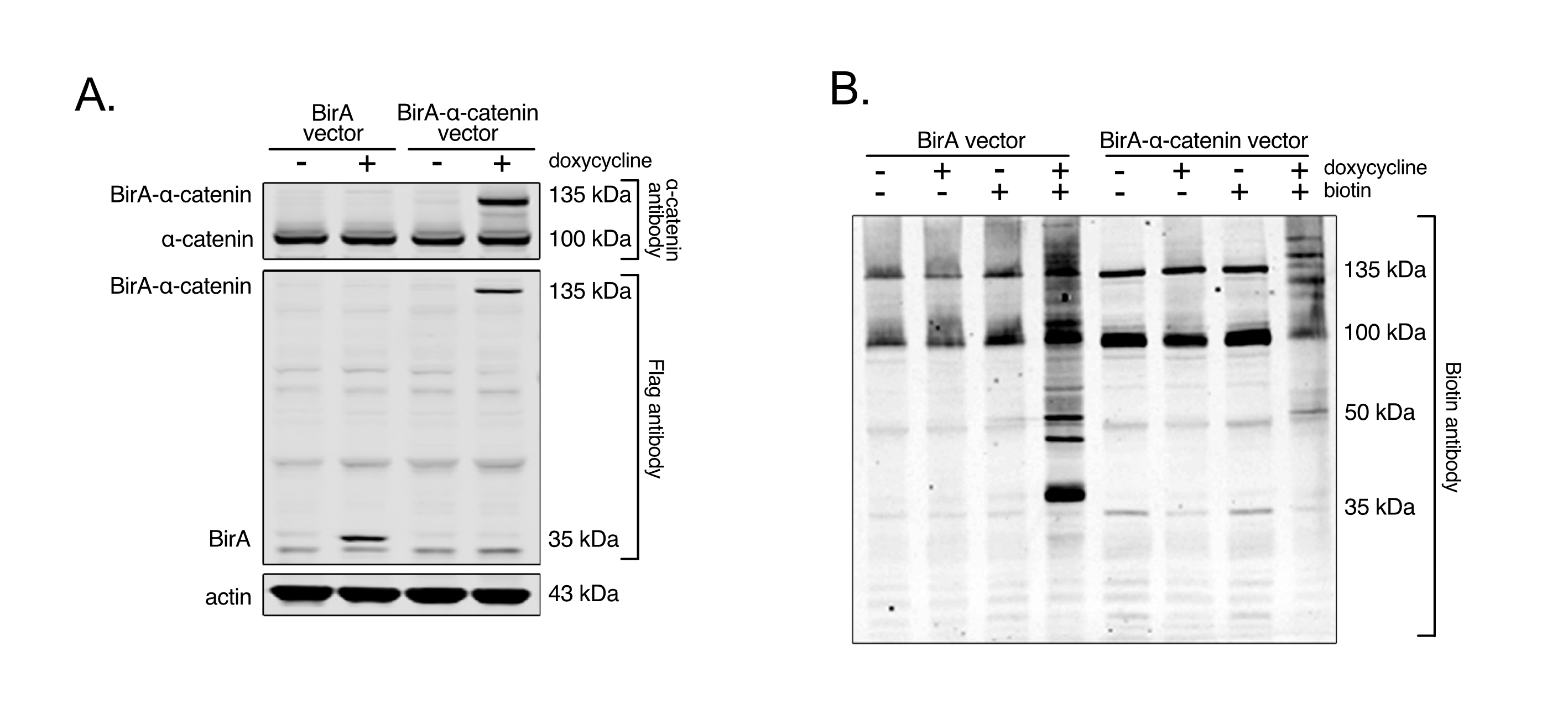

Supplement: Supplementary file 5 — Additional file 4: Figure S3. Identification of α-catenin binding partners using the BioID assay.Western immunoblot analysis illustrates the inducible expression of BirA-tagged α-catenin in HLF cells. A vector expressing only BirA was used as negative control.Combined treatment of cells with doxycycline and biotin for 24 h leads to biotinylation/laddering of proteins in cells expressing BirA or BirA-α-catenin. The intensity of laddering is weaker in cells with BirA-α-catenin expression compared to BirA expression alone, which is indicative for the specificity of the approach. [file 12964_2023_1169_MOESM4_ESM.tiff]

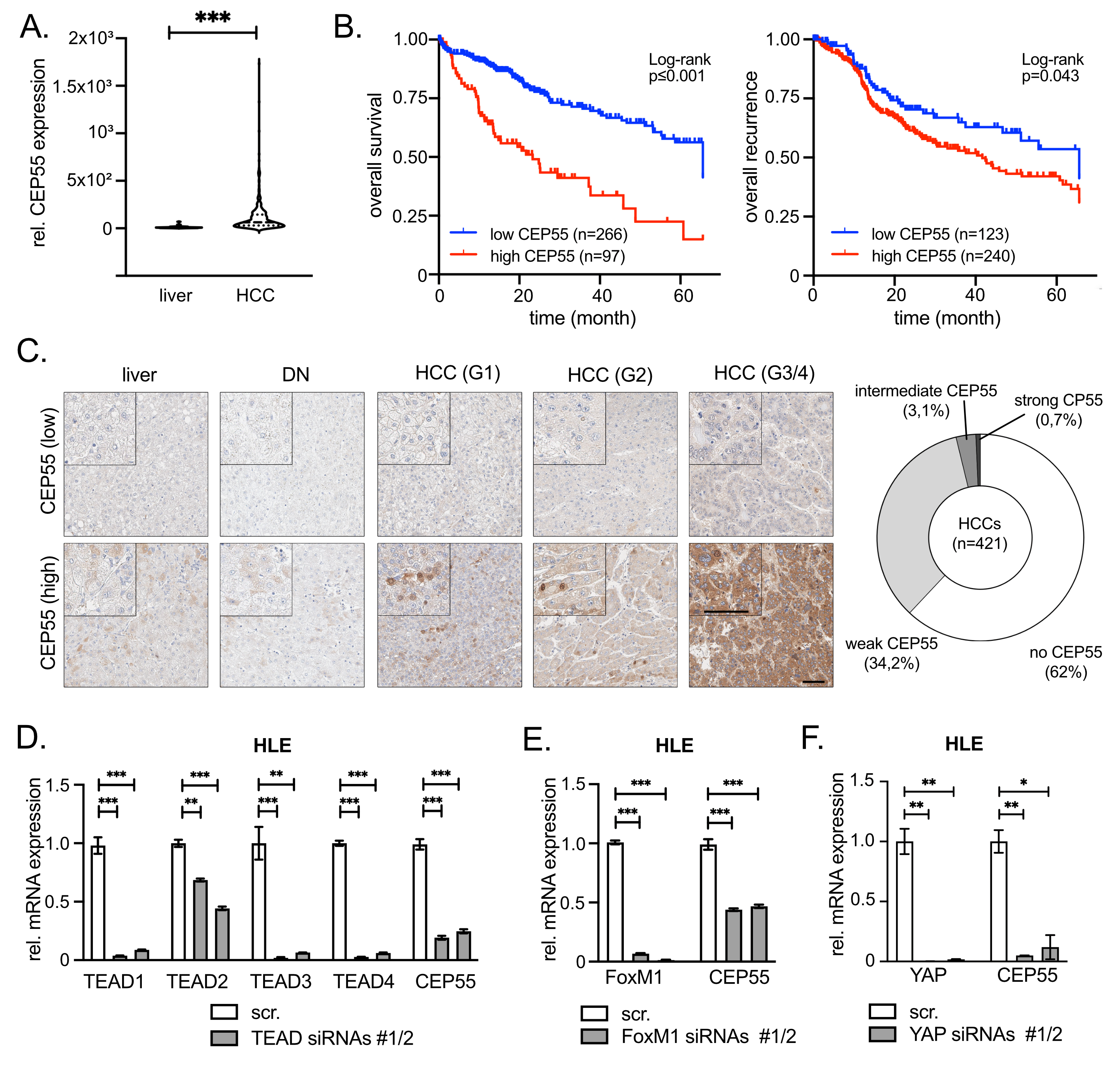

Supplement: Supplementary file 7 — Additional file 6: Figure S4. The TEADs/FoxM1/YAP complex regulates CEP55 in liver cancer cells.CEP55 expression analysis in an independent HCC patient cohort. Statistical test: Mann-Whitney U test. ***p≤0.001.Kaplan-Meier plots of overall patient survival and cancer recurrence according to low and high CEP55 expression [29]. Patients were divided in groups using Cutoff Finder. Statistical test: Log-rank test. p-values and group sizes are indicated.Representative images of immunohistochemistry stains for CEP55 with low and high expression. The pie chart illustrates the percentage of different CEP55 expression levels in the subgroup of HCC. The HCC tissue microarray contains normal livers, DNsand HCCs. Scale bars: 60 µm.Real-time PCR analysis of TEAD1-4 and CEP55, FoxM1 and CEP55, as well as YAP and CEP55after silencing of the respective transcriptional regulators by two siRNAsin HLE cells. For all RNAi experiments, scramblesiRNA-transfected cells served as controls. Statistical test: Mann-Whitney U test. *p≤0.05, **p≤0.01, ***p≤0.001. [file 12964_2023_1169_MOESM6_ESM.tiff]

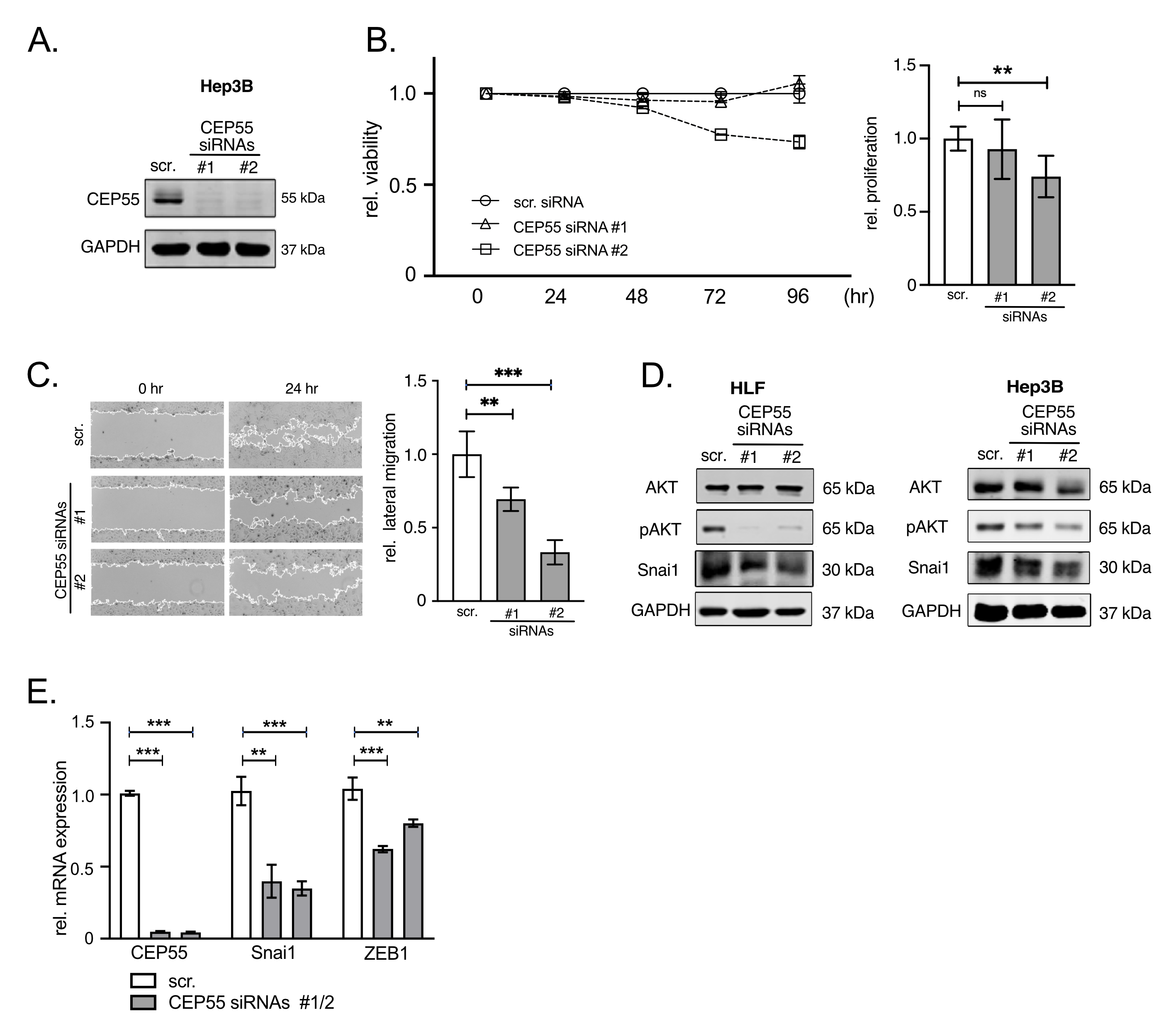

Supplement: Supplementary file 8 — Additional file 7: Figure S5. CEP55 supports HCC cell migration but not proliferation.Western immunoblot for CEP55 in Hep3B cells after transfection of two gene-specific siRNAs. Samples were isolated 48 h after transfection.Cell viability assay and proliferation assay of Hep3B cells after siRNA-mediated silencing of CEP55. Viability was measured at indicated timepoints and proliferation was detected after 72 h.Lateral migration of Hep3B cells after CEP55 silencing was detected using a 'scratch' assay after 24 h. Cells were pretreated with mitomycin-C.Western immunoblot after CEP55 silencing in HLF and Hep3B cells. The expression of AKT, its phosphorylation, and the expression of the EMT-related protein Snai1were analyzed.Real-time PCR analysis of the EMT genes Snai1 and ZEB1 after RNAi-mediated CEP55 inhibition. Statistical test used in,, and: Mann-Whitney U test. **p≤0.01, ***p≤0.001. [file 12964_2023_1169_MOESM7_ESM.tiff]
