# Supplementary material for: α-catenin interaction with YAP/FoxM1/TEAD-induced CEP55 supports liver cancer cell migration
Source: Cell Commun Signal. 2023 Jun 28;21:162. doi: 10.1186/s12964-023-01169-2 (PMC10304383; doi:10.1186/s12964-023-01169-2)
Supplement: Supplementary file 6 — Additional file 5: Suppl. Table 7. Potential α‐catenin binding partners. [file 12964_2023_1169_MOESM5_ESM.docx]

**α-catenin interaction with YAP/FoxM1/TEAD-induced CEP55 supports liver cancer cell migration**

Y. Tang, L. Thiess, S.M.E. Weiler, M. Tóth, F. Rose, S. Merker, T. Ruppert, P. Schirmacher, K. Breuhahn

**Suppl. Table 7: Potential α‐catenin binding partners**

| Gene name | Protein ID | Fold change | p-value | Protein function |
| --- | --- | --- | --- | --- |
| ABLIM1 | O14639 | 7.61 | p≤0.05 | scaffold protein/actin binding |
| ANK3 | Q12955 | 7.75 | p≤0.05 | membrane-cytoskeleton linker/actin binding |
| CAT | P04040 | 3.60 | p=0.37 | antioxidant enzyme |
| CD44 | P16070 | 3.38 | p=0.074 | cell-cell interactions |
| CEP55 | Q53EZ4 | 3.86 | p=0.059 | mitosis and cytokinesis, migration |
| CLMN | Q96JQ2 | 4.34 | p≤0.05 | actin binding |
| CTNND1 | O60716 | 1585 | p≤0.05 | cell-cell adhesion |
| CYFIP1 | Q7L576 | 3.52 | p≤0.05 | actin binding and dynamics |
| ECD | O95905 | 15.94 | p≤0.05 | acetyltransferase/transcriptional regulation |
| EGFR | P00533 | 3.98 | p=0.068 | tyrosine kinase receptor |
| ERBB2IP | Q96RT1 | 50.69 | p≤0.05 | signaling pathway regulator |
| FERMT2 | Q96AC1 | 3.86 | p≤0.05 | scaffold protein/actin binding |
| JUP | P14923 | 11.89 | p≤0.05 | junctional protein |
| LPP | Q93052 | 73.30 | p≤0.05 | cell adhesion, transcriptional coactivator |
| MAP4K4 | O95819 | 4.30 | p≤0.05 | signaling pathway regulator |
| MLLT4 | P55196 | 43.51 | p≤0.05 | junctional protein |
| NAP1L1 | P55209 | 5.17 | p≤0.05 | DNA replication |
| NAP1L4 | Q99733 | 5.24 | p≤0.05 | nucleosome assembly |
| NUMB | P49757 | 5.83 | p≤0.05 | histone modulator |
| OCLN | Q16625 | 4.79 | p≤0.05 | junctional protein |
| PARD3 | Q8TEW0 | 8.64 | p≤0.05 | cell polarity |
| PDLIM7 | Q9NR12 | 11.15 | p≤0.05 | scaffold protein/ actin binding |
| PHACTR4 | Q8IZ21 | 6.90 | p≤0.05 | phosphatase inhibitor/actin binding |
| PLEKHA5 | Q9HAU0 | 79.13 | p≤0.05 | metabolism |
| PTPN13 | Q12923 | 4.44 | p≤0.05 | mitosis, phosphatase |
| SCRIB | Q14160 | 90.12 | p≤0.05 | scaffold protein, cell polarity |
| SEPT2 | Q15019 | 4.74 | p=0.051 | septin cytoskeleton |
| SNAP23 | O00161 | 4.55 | p≤0.05 | vesicular transport |
| SPTBN1 | Q01082 | 3.56 | p=0.088 | scaffold protein/ actin binding |
| STEAP3 | Q658P3 | 4.64 | p≤0.05 | iron transporter |
| TJP1 | Q07157 | 15.03 | p≤0.05 | scaffold protein, junctional protein |
| TJP2 | Q9UDY2 | 3.98 | p=0.061 | scaffold protein, junctional protein |
| TXNL1 | O43396 | 4.19 | p=0.055 | reductase |
| YKT6 | O15498 | 3.79 | p=0.103 | vesicular transport |
