# Supplementary material for: α-catenin interaction with YAP/FoxM1/TEAD-induced CEP55 supports liver cancer cell migration
Source: Cell Commun Signal. 2023 Jun 28;21:162. doi: 10.1186/s12964-023-01169-2 (PMC10304383; doi:10.1186/s12964-023-01169-2)
Supplement: Supplementary file 9 — Additional file 8. [file 12964_2023_1169_MOESM8_ESM.pdf]

α-catenin interaction with YAP/FoxM1/TEAD-induced CEP55 supports liver cancer cell migration

Y. Tang et al - raw image files

Figure 2A

α-catenin

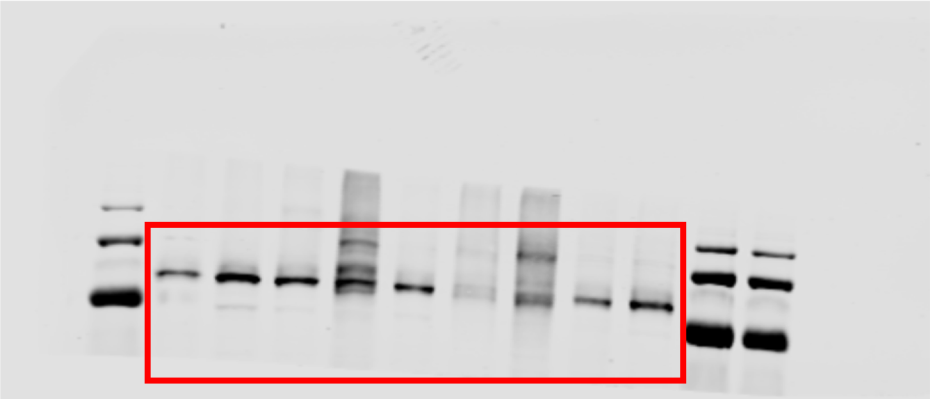

GAPDH

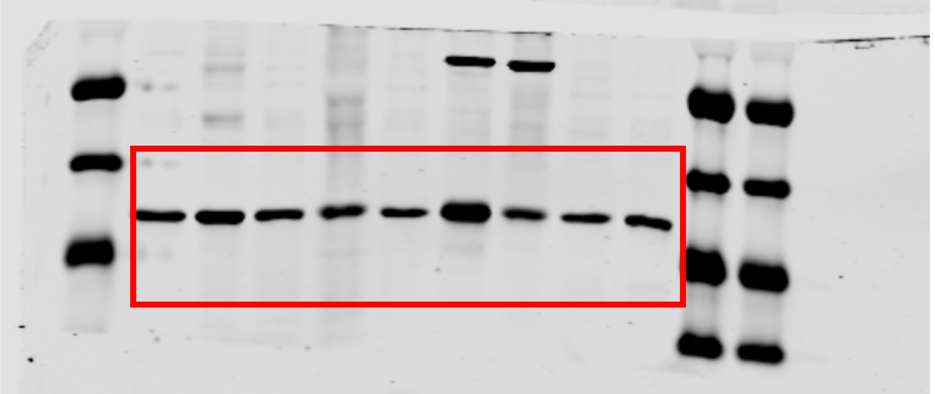

Figure 2C

α-catenin

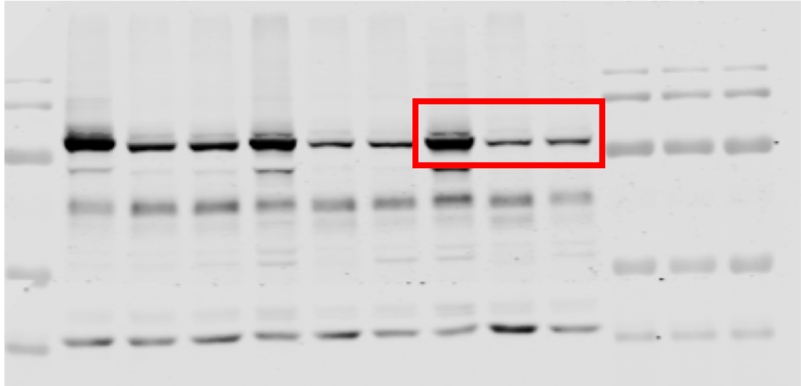

actin

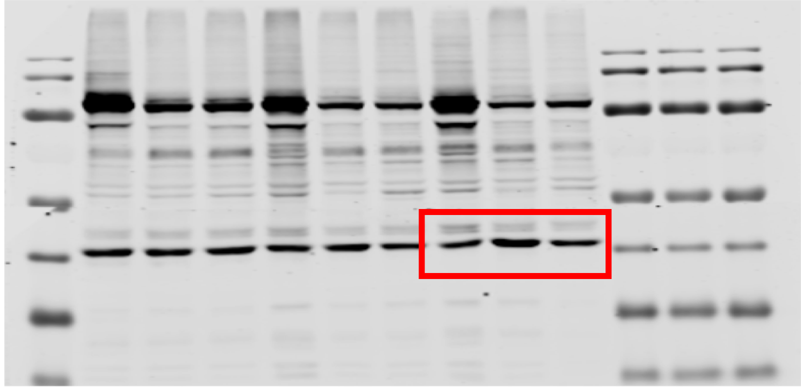

Figure 2: Functional relevance of α-catenin in hepatocarcinogenesis.

(A.) Western blot analysis detecting α-catenin in human liver cancer cell line lysates (n=8) and the immortalized human hepatocyte cell line HHT4.

(C.) Representative Western immunoblot and qPCR of α-catenin in HLF cells after transfection of two independent α-catenin-specific siRNAs (#1, #2). Samples were isolated 72 hr after transfection.

α-catenin interaction with YAP/FoxM1/TEAD-induced CEP55 supports liver cancer cell migration

Y. Tang et al - raw image files

Figure 3D

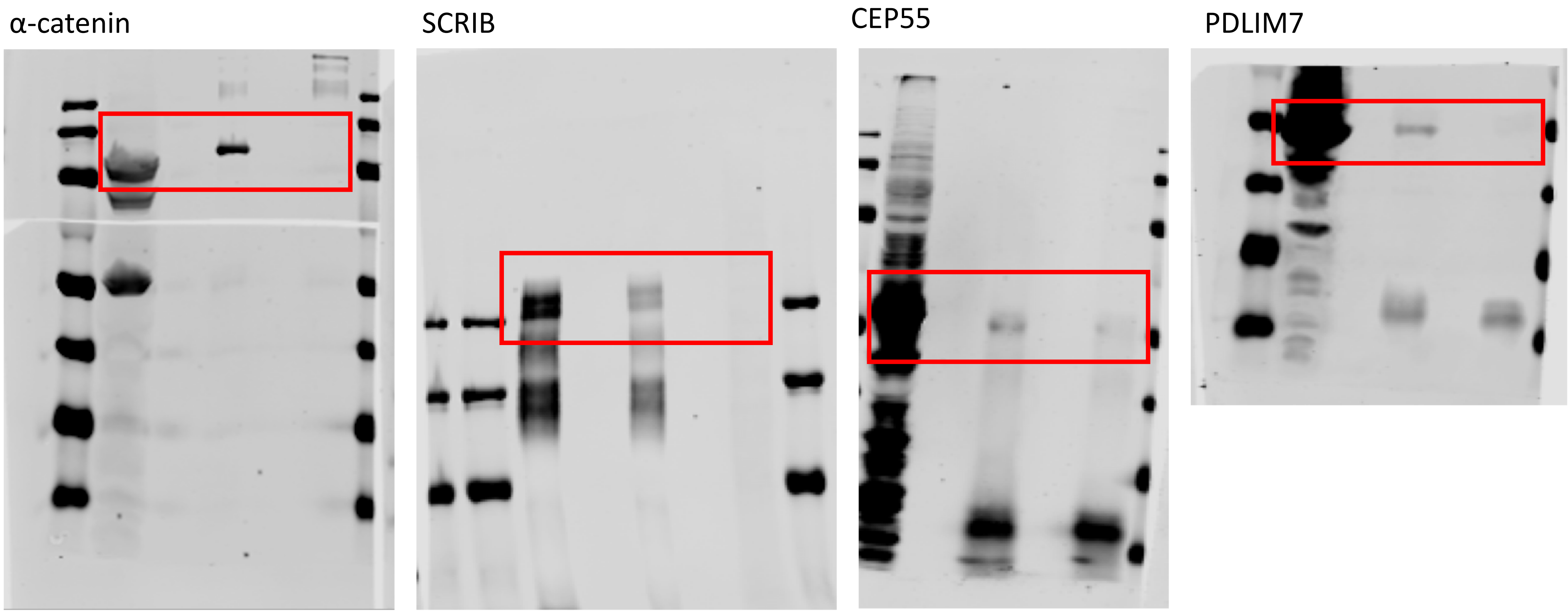

Figure 3. CEP55 and PDLIM7 bind α-catenin in the cytoplasm of HCC cells.

(D.) Co-IP utilizing cell extracts from HLF cells transfected with Flag-tagged human α-catenin followed by detection of α-catenin, CEP55, or PDLIM7. SCRIB, which is a known interaction partner of the cadherin/catenin complex, was used as positive control.

α-catenin interaction with YAP/FoxM1/TEAD-induced CEP55 supports liver cancer cell migration

Y. Tang et al - raw image files

Figure 4D

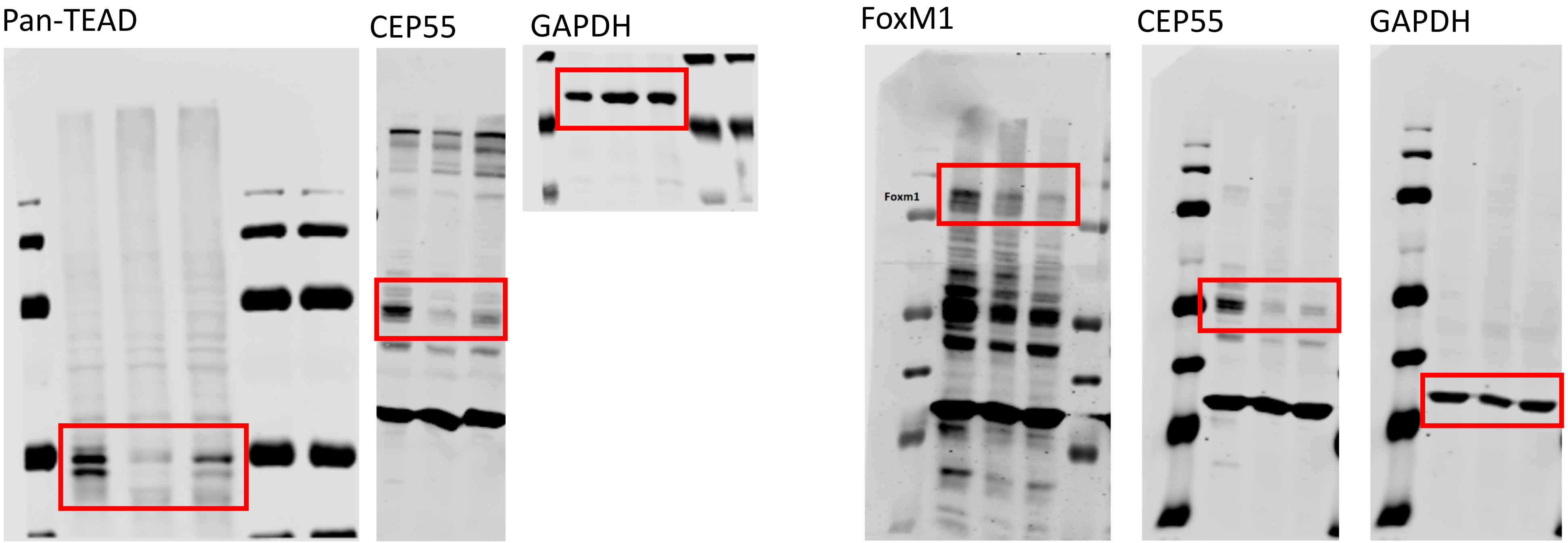

Figure 4. The TEADs/FoxM1/YAP complex regulates CEP55 overexpression in liver cancer cells.

(D.) Western Immunoblot data of HLF cells after gene-specific silencing of TEAD1/3/4 family members, FoxM1, and YAP for 48 hr. Due to structural differences, TEAD2 is not efficiently targeted by the chosen siRNAs.

α-catenin interaction with YAP/FoxM1/TEAD-induced CEP55 supports liver cancer cell migration

Y. Tang et al - raw image files

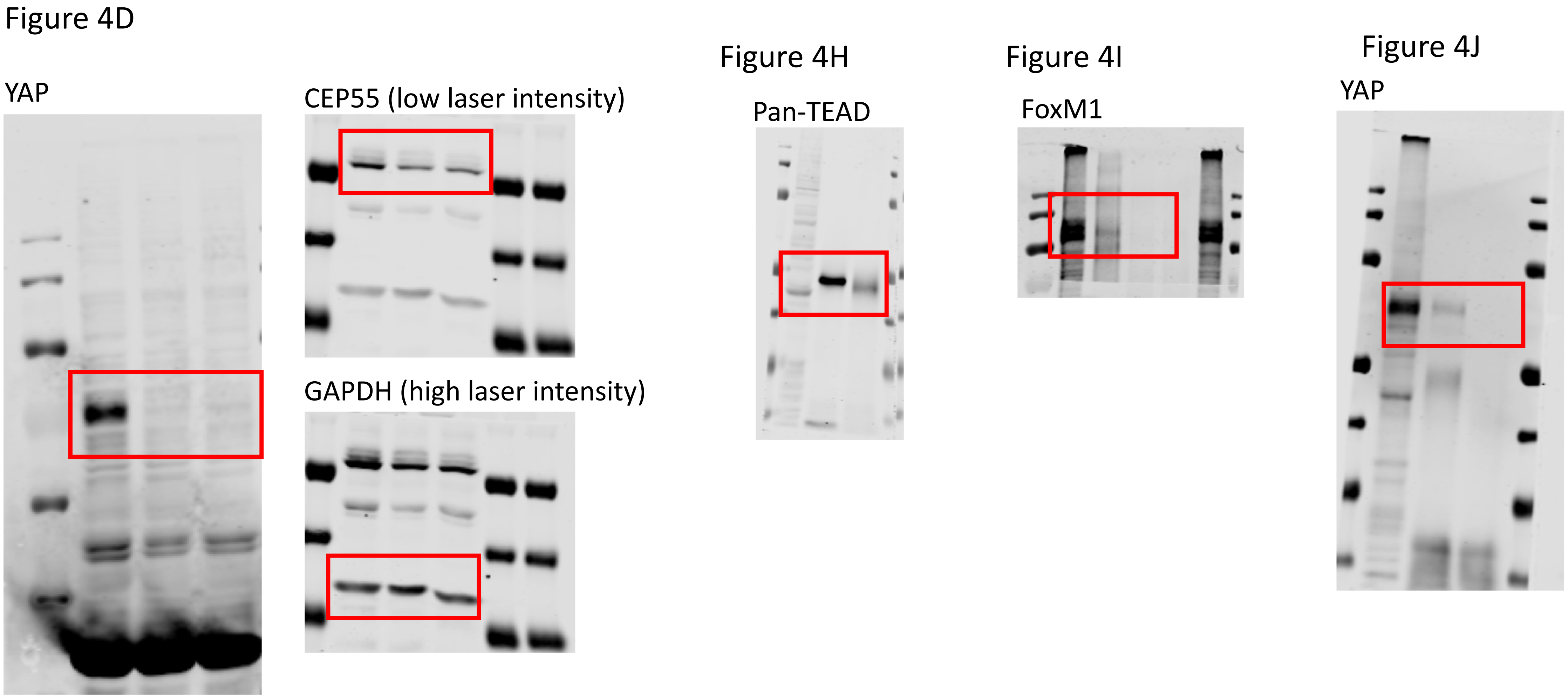

Figure 4. The TEADs/FoxM1/YAP complex regulates CEP55 overexpression in liver cancer cells.

(D.) Western Immunoblot data of HLF cells after gene-specific silencing of TEAD1/3/4 family members, FoxM1, and YAP for 48 hr. Due to structural differences, TEAD2 is not efficiently targeted by the chosen siRNAs.

(H.-J.) ChIP analysis of TEAD4 **(H.)**, FoxM1 **(I.)**, and YAP **(J.)** at two predicted binding sites (BS) in the CEP55 promoter (BS#1 and BS#2). Two CEP55 upstream promoter regions served as negative controls (BS#1 and BS#2). IgG was employed as antibody control. Results were normalized to respective IgG controls. Western blots in H.-J. illustrate successful IP of TEADs, FoxM1, and YAP.

α-catenin interaction with YAP/FoxM1/TEAD-induced CEP55 supports liver cancer cell migration

Y. Tang et al - raw image files

Figure 5A

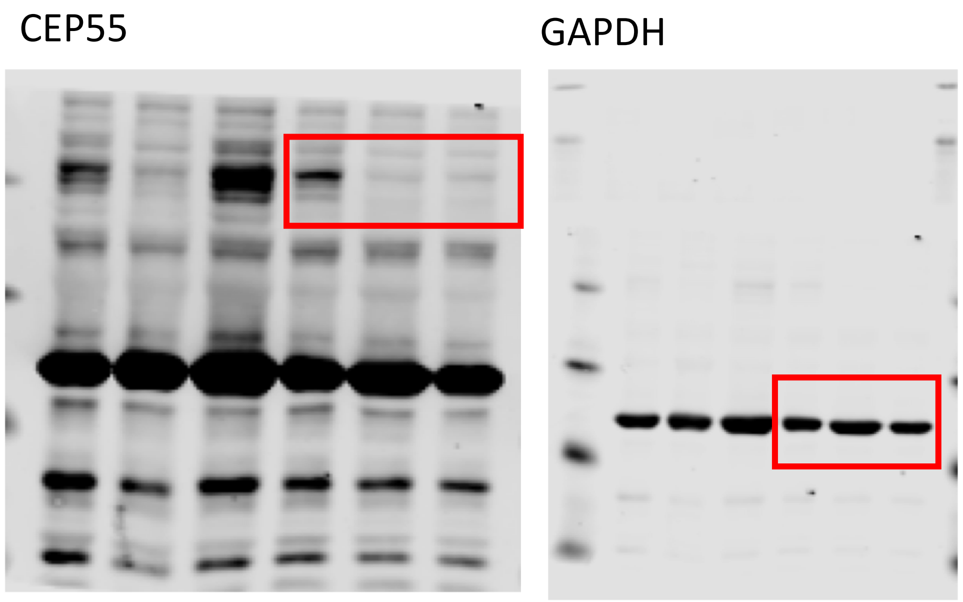

Figure 5E

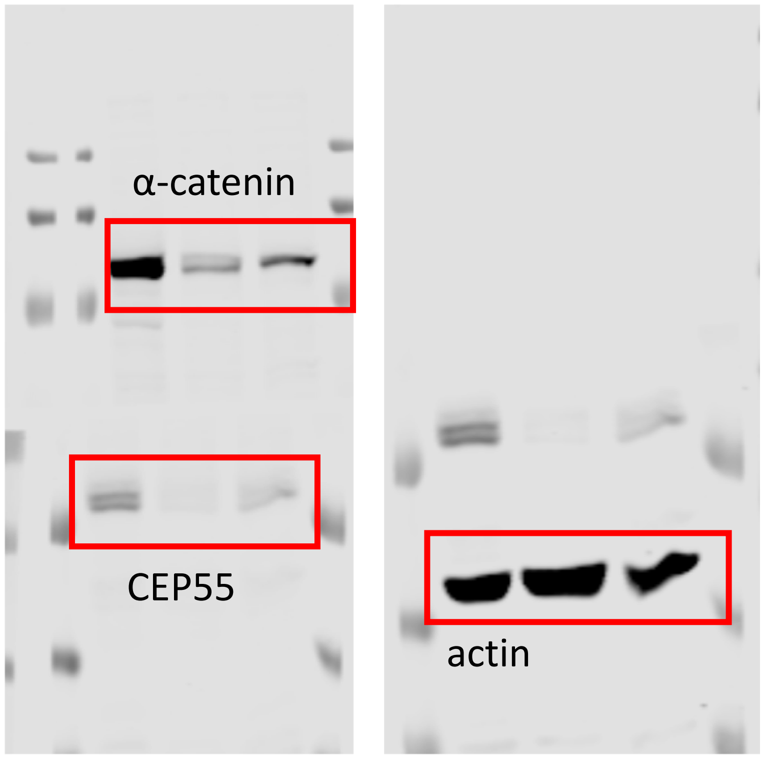

Figure 5. α-catenin stabilizes CEP55 to support HCC cell migration but not proliferation.

(A.) Western immunoblot of CEP55 in HLF cells after transfection of two independent gene-specific siRNAs (#1, #2). Samples were isolated 48 hr after transfection.

(E.) Western immunoblot after inhibition of α-catenin by siRNAs in HLF cells. Samples were isolated 72 hr after transfection.

α-catenin interaction with YAP/FoxM1/TEAD-induced CEP55 supports liver cancer cell migration

Y. Tang et al - raw image files

Suppl. Figure 2A

HepG2 (high laser power)

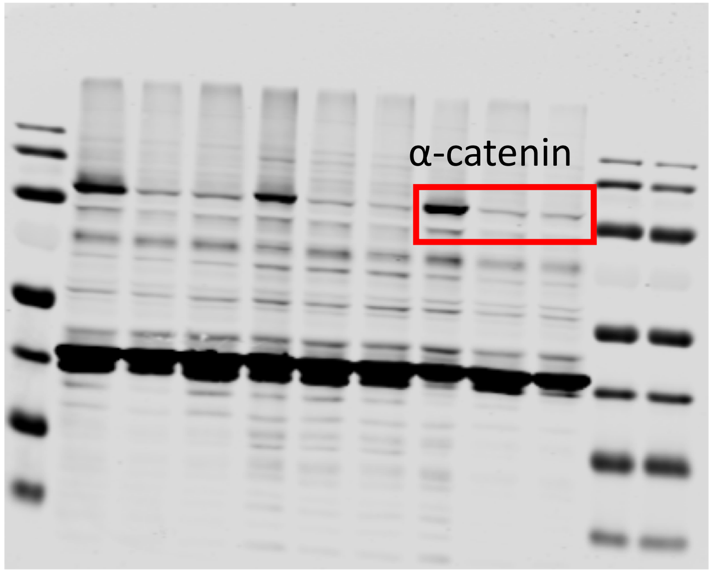

HepG2 (low laser power)

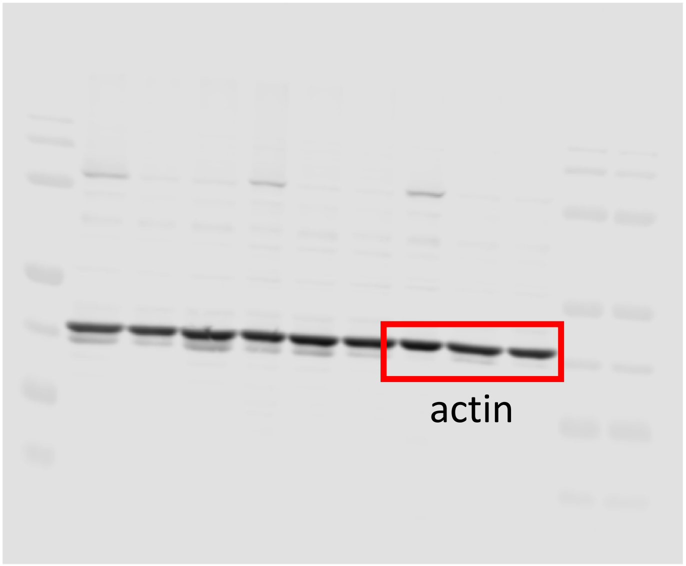

Hep3B

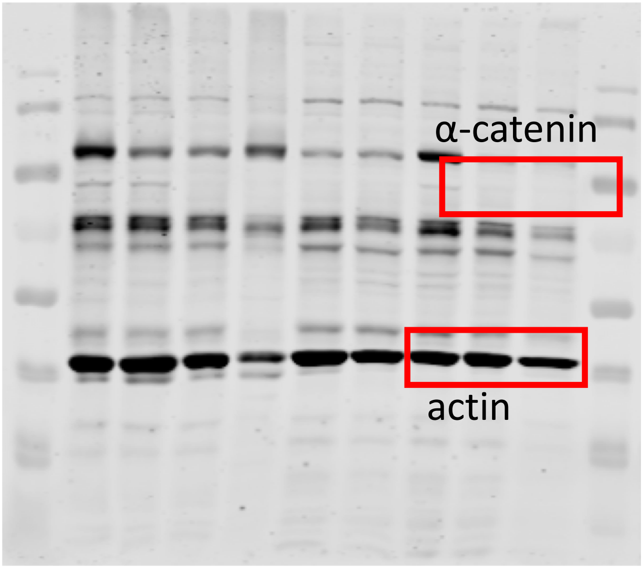

Figure S2: Functional relevance of α-catenin in hepatocarcinogenesis.

(A.) Representative Western immunoblot for α-catenin in HepG2 and Hep3B cells after transfection of two α-catenin-specific siRNAs (#1, #2). Samples were isolated 72 hr after transfection.

α-catenin interaction with YAP/FoxM1/TEAD-induced CEP55 supports liver cancer cell migration

Y. Tang et al - raw image files

Suppl. Figure 3A

α-catenin / BirA-α-catenin

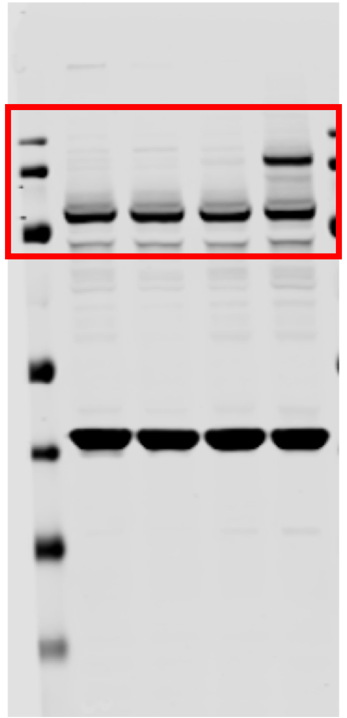

Flag

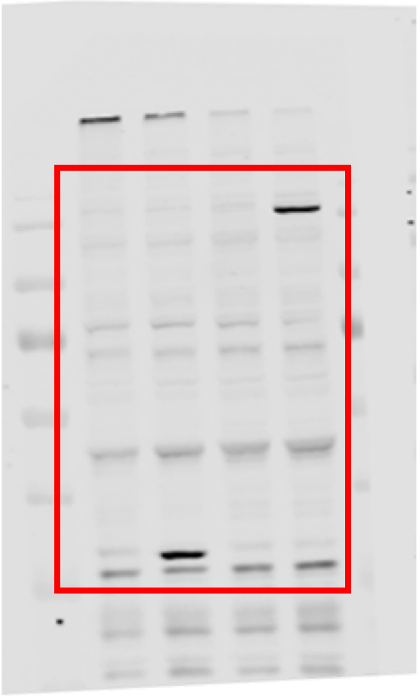

Suppl. Figure 3B

Biotin

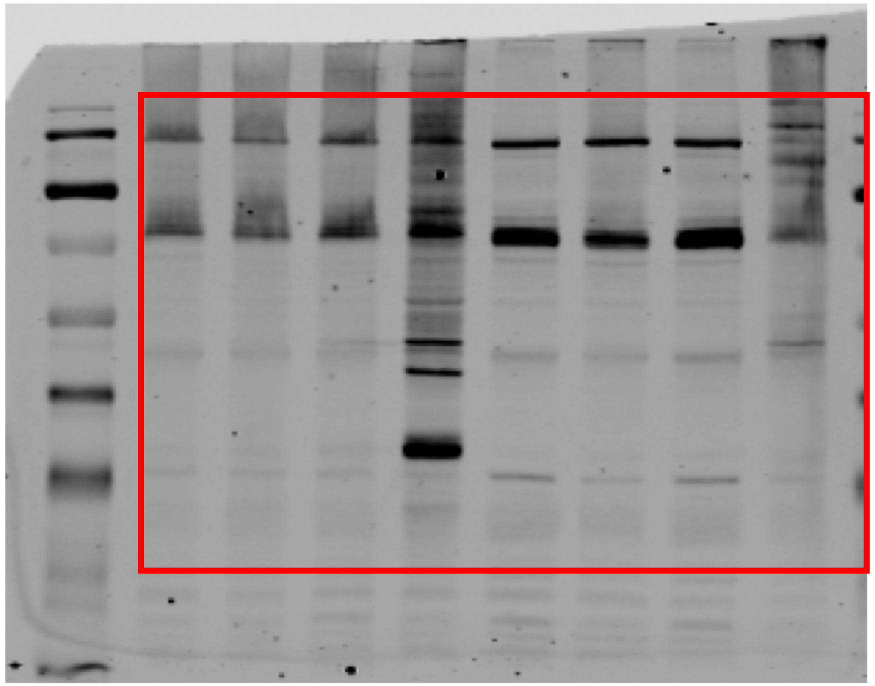

Figure S3: Identification of α-catenin binding partners using the BioID assay.

(A.) Western immunoblot analysis illustrates the inducible expression of BirA-tagged α-catenin in HLF cells (135 kDa). A vector expressing only BirA was used as negative control (BirA vector; 35 kDa).

α-catenin interaction with YAP/FoxM1/TEAD-induced CEP55 supports liver cancer cell migration

Y. Tang et al - raw image files

Suppl. Figure 5A

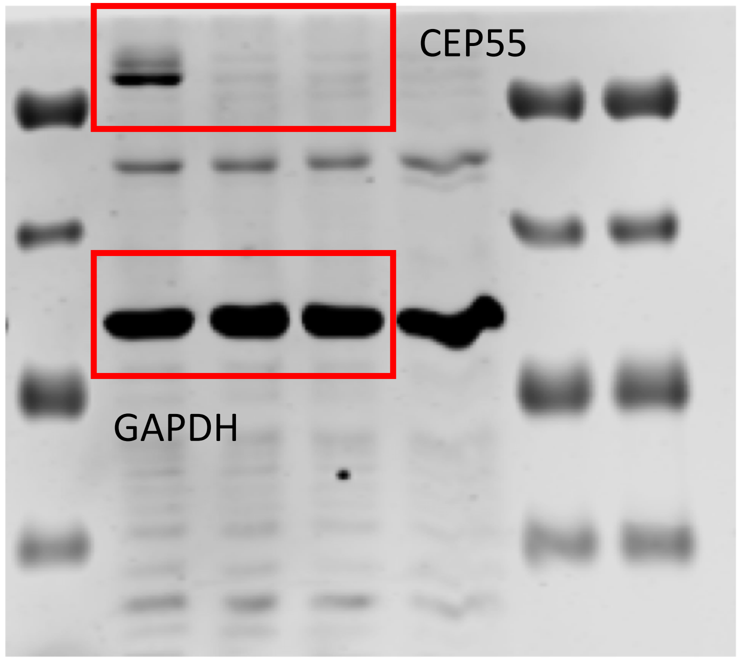

Suppl. Figure 5D

HLF

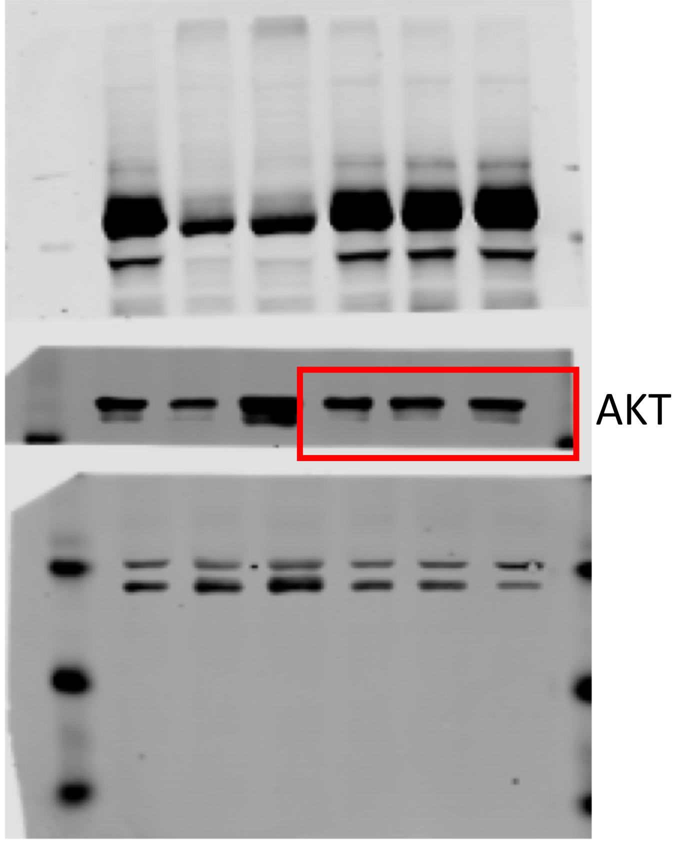

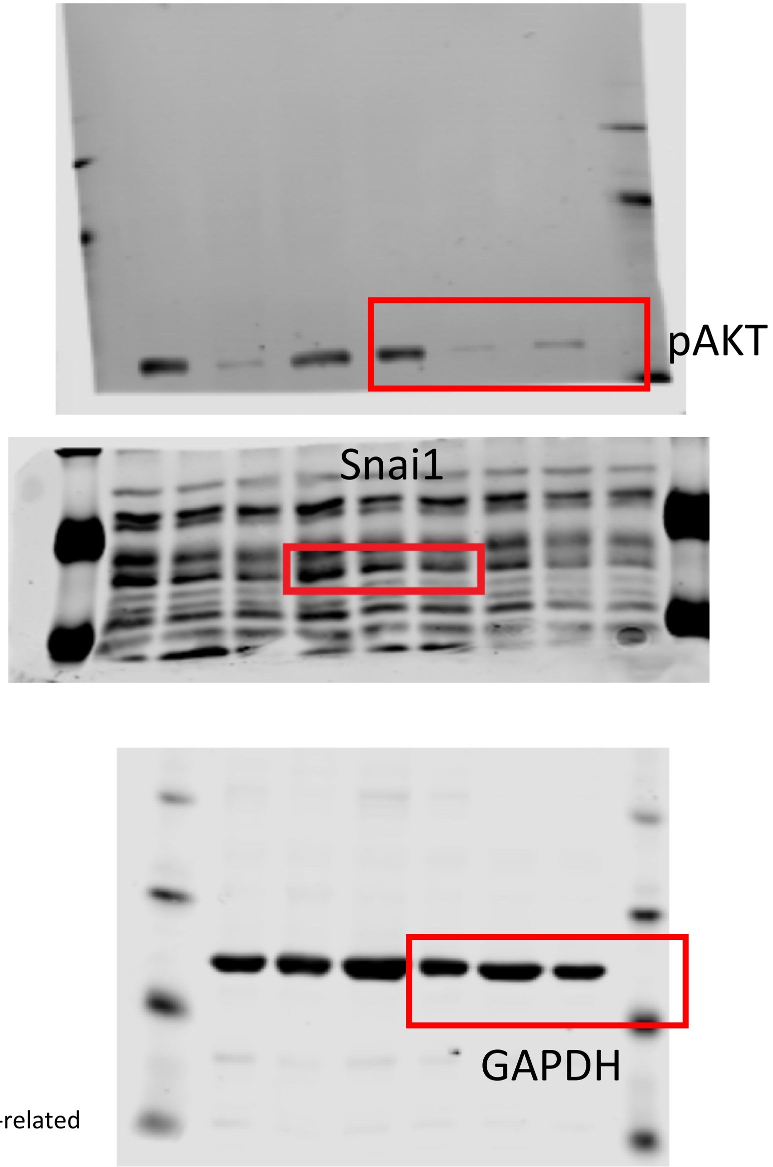

Figure S5: CEP55 supports HCC cell migration but not proliferation.

(A.) Western immunoblot for CEP55 in Hep3B cells after transfection of two gene-specific siRNAs (#1, #2). Samples were isolated 48 hr after transfection.

(D.) Western immunoblot after CEP55 silencing in HLF and Hep3B cells. The expression of AKT, its phosphorylation (pAKT), and the expression of the EMT-related protein Snai1 (synonyms: SNAIL, SNAIL1) were analyzed.

α-catenin interaction with YAP/FoxM1/TEAD-induced CEP55 supports liver cancer cell migration

Y. Tang et al - raw image files

Suppl. Figure 5D

Hep3B

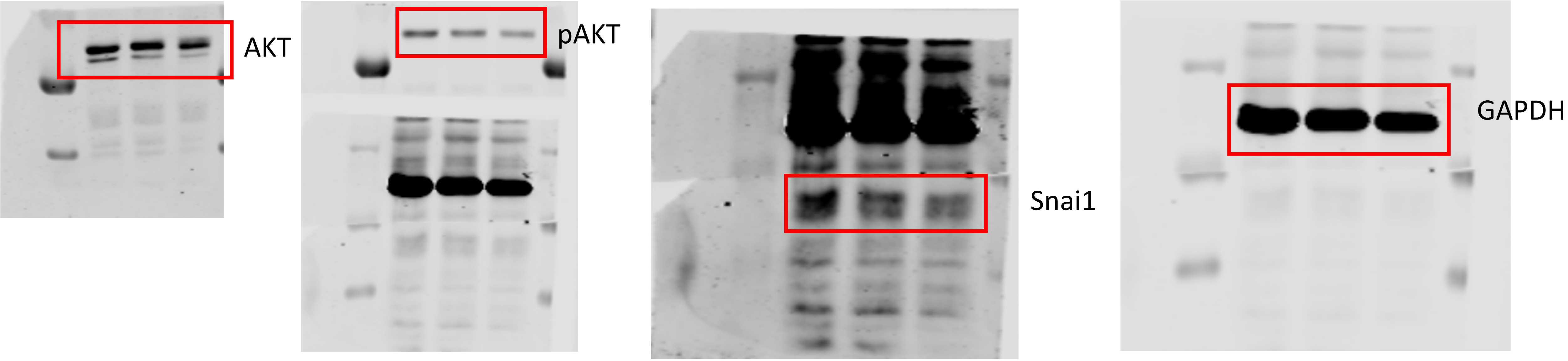

Figure S5: CEP55 supports HCC cell migration but not proliferation.

(D.) Western immunoblot after CEP55 silencing in HLF and Hep3B cells. The expression of AKT, its phosphorylation (pAKT), and the expression of the EMT-related protein Snai1 (synonyms: SNAIL, SNAIL1) were analyzed.
